# Atomic defect classification of the H–Si(100) surface through multi-mode scanning probe microscopy

**DOI:** 10.3762/bjnano.11.119

**Published:** 2020-09-07

**Authors:** Jeremiah Croshaw, Thomas Dienel, Taleana Huff, Robert Wolkow

**Affiliations:** 1 Department of Physics, University of Alberta, Edmonton, Alberta, T6G 2J1, Canada; 2 Quantum Silicon, Inc., Edmonton, Alberta, T6G 2M9, Canada; 3 Department of Materials Science and Engineering, Cornell University, Ithaca NY 14853, USA; 4 Nanotechnology Research Centre, National Research Council Canada, Edmonton, Alberta, T6G 2M9, Canada

**Keywords:** atomic force microscopy, hydrogen-terminated silicon, scanning tunnelling hydrogen microscopy, scanning tunnelling microscopy, surface metrology

## Abstract

The combination of scanning tunnelling microscopy (STM) and non-contact atomic force microscopy (nc-AFM) allows enhanced extraction and correlation of properties not readily available via a single imaging mode. We demonstrate this through the characterization and classification of several commonly found defects of the hydrogen-terminated silicon (100)-2 × 1 surface (H–Si(100)-2 × 1) by using six unique imaging modes. The H–Si surface was chosen as it provides a promising platform for the development of atom scale devices, with recent work showing their creation through precise desorption or placement of surface hydrogen atoms. While samples with relatively large areas of the H–Si surface are routinely created using an in situ methodology, surface defects are inevitably formed reducing the area available for patterning. By probing the surface using the different interactivity afforded by either hydrogen- or silicon-terminated tips, we are able to extract new insights regarding the atomic and electronic structure of these defects. This allows for the confirmation of literature assignments of several commonly found defects, as well as proposed classifications of previously unreported and unassigned defects. By combining insights from multiple imaging modes, better understanding of their successes and shortcomings in identifying defect structures and origins is achieved. With this, we take the first steps toward enabling the creation of superior H–Si surfaces through an improved understanding of surface defects, ultimately leading to more consistent and reliable fabrication of atom scale devices.

## Introduction

Novel approaches to advance integrated circuitry beyond CMOS have focused on atom scale structures and their reliable fabrication [1]. Hydrogen-terminated silicon (H–Si) surfaces are one such versatile platform for the patterning and operation of atom scale devices including qubits [2–3] and single electron transistors [4–5] made from atomically precise implanted donor atoms near the H–Si surface, and logic structures using fabricated silicon dangling bonds [6–8]. In many cases the structures’ functional elements are comprised of a few or even single atoms. At such dimensions, atomic scale defects of the surface and in the shallow subsurface region can have a significant impact on device patternability and operation [9]. In order to develop suitable means to accommodate defects, whether it be by optimizing sample preparation, quantifying how defects affect device operation [9], or by using convolutional neural networks to autonomously identify defects [10–12], a comprehensive understanding of the many varieties of defects is needed.

Native silicon atoms at the unreconstructed (unterminated) (100) surface would, by argument of the crystal geometry, extend two unsatisfied bonds into vacuum. To minimize the surface energy, each silicon atom bonds with a neighbouring Si atom to create a dimer, thus reducing the number of dangling bonds (DBs) by half [13]. Rows consisting of many of these dimers are formed, which run parallel along the surface. The study of Si(100) surface defects was one of the first applications of scanning probe microscopy [14]. The three observed species were identified as a missing silicon dimer, a pair of missing silicon dimers, and a missing pair of Si atoms on the same side of two neighbouring dimers. Subsequently, the latter had been reassigned as an H, OH pair originating from dissociative attachment of a residual water molecule in the vacuum system [15–17]. Further insights became available by non-contact atomic force microscopy (nc-AFM), separating the electronic and structural behaviour of the Si(100) surface [18].

**Figure 1:**
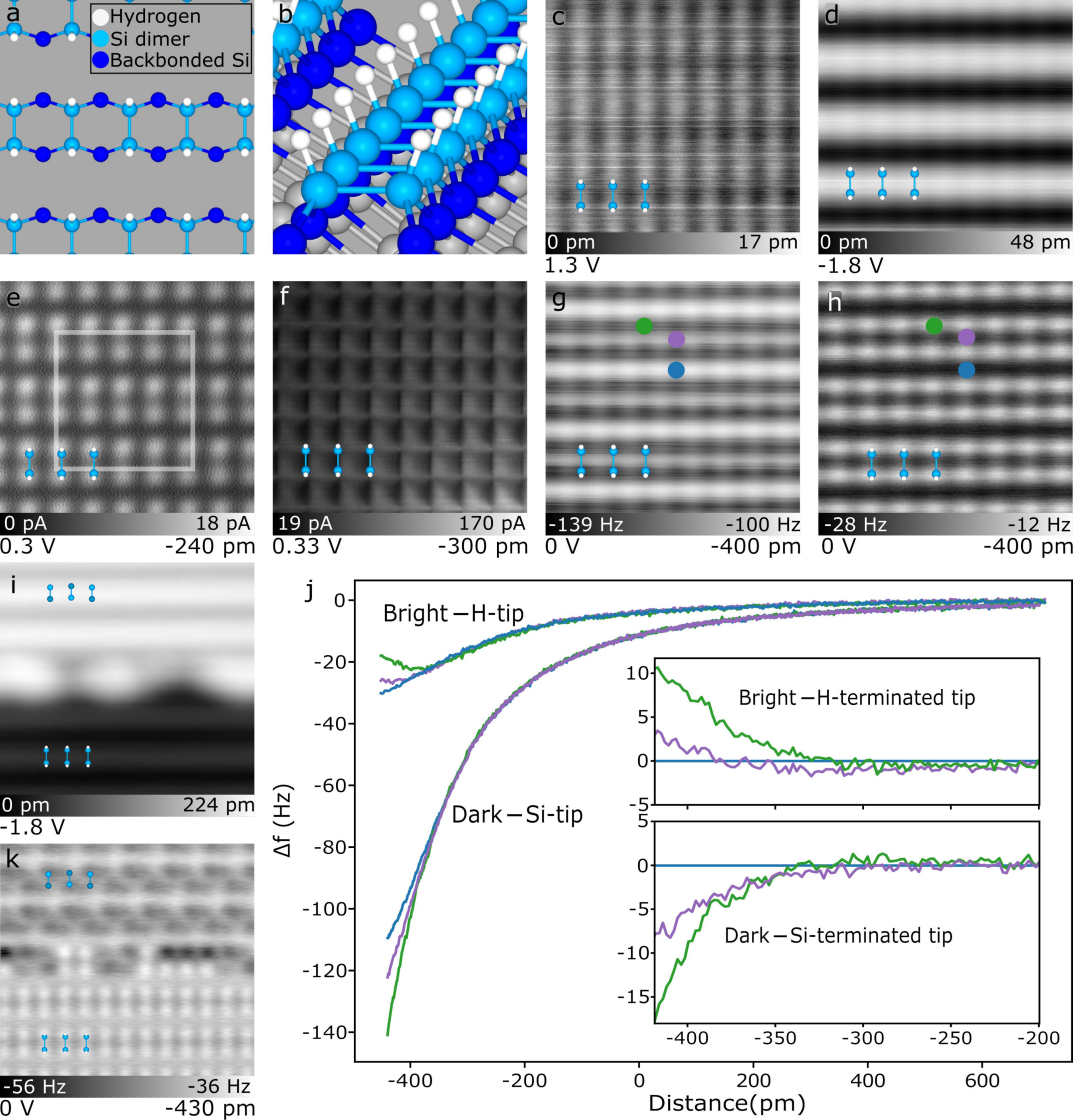
Surface of H-terminated Si(100)-2 × 1 in different imaging modes. (a,b) Top and isometric projection of a structural model of the H-terminated Si(100)-2 × 1 surface. The size of the area shown in (a) is outlined in (e). (c,d) Constant current (*I* = 50 pA) STM topography probing empty and filled states of the surface (bias voltages indicated in the lower left of each panel). (e) Constant height STM image with a fixed bias and height. (f) Scanning tunnelling hydrogen microscopy image of the surface. (g) nc-AFM frequency shift map using a Si-functionalized tip that displays the hydrogen atoms in “dark contrast”. (h) nc-AFM frequency shift map measured with a H-functionalized tip showing hydrogen atoms in “bright contrast”. Images (c–h) are all 3.2 × 3.2 nm^2^, the indicated height offset (Δ*z* at the bottom right of relevant panels) is initialized referencing a STM tunnelling setpoint of *I* = 50 pA and *V* = −1.8 V over a H–Si surface atom. (i) Constant current STM topography of a half hydrogen-terminated (bottom) half unterminated silicon (top) surface (5 × 5 nm^2^, *V* = −1.8 V, *I* = 50 pA). (j) Position-dependent frequency shift spectroscopy Δ*f*(*z*) in bright and dark nc-AFM modes (positions indicated in (g) and (h)), highlighting the quantitative differences in tip reactivity for different apex terminations. The insets show the calculated difference spectra in reference to the spectra taken between the dimer rows (blue position marker). (k) AFM image of the same area shown in (i) using a H-functionalized, bright contrast tip. The bottom part shows the characteristic bright contrast features of H–Si, while the unterminated Si portion shows the inverted contrast (atoms appear dark) of alternatingly buckled, unterminated dimers with a slight double tip artefact.

The addition of hydrogen to surface silicon atoms saturates all available bonds [19] and three surface reconstructions are commonly observed. The 2 × 1 phase – frequently used in hydrogen lithography, and can be prepared in situ resulting in large, defect free areas [20] – has each surface Si atom in a dimer bonded to one hydrogen atom (Figure 1a,b). The 1 × 1 phase is characterized by the absence of dimer bonds, with each surface Si atom instead saturated by 2 hydrogen atoms, forming silicon dihydrides (H_2_-Si). The 3 × 1 phase is a combination of the previous two, consisting of alternating 2 × 1 dimers and 1 × 1 dihydrides [21–22]. With continued study, it became apparent that the complexity of possible surface reconstructions and surface defects extended well beyond those initially observed. Here, we provide a comprehensive overview of the H-terminated Si(100)-2 × 1 surface, its structural features, and defects. Six different scanning probe imaging modes are performed using both STM and nc-AFM. By combining the accessible information with probe particle simulations [23–24] (presented in Supporting Information File 1) of the expected structural geometry (see section Methods), we are able to confirm the atomic structure of several commonly reported defects, as well as to classify previously unknown defects. The latter includes a point defect that can be found decorated with a single H-atom, rendering the otherwise neutral structure negatively charged. We demonstrate the tip-induced removal of the weakly bound H atom, leaving the site neutral. While we present an extensive experimental analysis supplemented with simple simulations of common surface defects on H-terminated silicon, we anticipate that the provided assignments will foster further investigation with more robust theoretical frameworks such as by density functional theory.

## Results and Discussion

### SPM imaging modes

The variability observed in differing scanning probe imaging modes originates from the applied feedback mode, different tunnelling parameters, or the functionalization of the probe tip. Figure 1 showcases the imaging modes employed in this work as applied to the defect-free H–Si(100)-2 × 1 surface (see Methods for details about the sample preparation). In the well-known STM topographies probing empty and filled states in Figure 1c and 1d, respectively, the dimer rows can readily be seen running horizontally across the image (constant current imaging [50 pA], sample bias as indicated in the lower left of each panel). Recent work has reported that tip functionalization affects the contrast sharpness and apparent atomic positions in STM images of the H–Si(100) surface [25–26]. Additional examples of this effect are displayed in Supporting Information File 1, Figure S1.

Constant height STM images can provide further insights, as shown in Figure 1e, probing the onset of the conduction band and donor band of our crystal (sample bias: +0.3 V) [9,18,27]. Individual atoms within each dimer are clearly visible, with minimal conductance occurring directly through the bulk states (requiring a reduction in tip–sample separation). This imaging mode is useful for cases where excessive bulk current could mask more subtle features. For all constant height modes, the reported approach of the tip is relative to a constant current tunnelling position above an H–Si atom with a sample bias of −1.8 V and a tunnelling current of 50 pA (as discussed in Methods). The heights for each mode are chosen to optimize the desired surface contrast (as shown in Supporting Information File 1, Figure S2) while avoiding unwanted tip–sample contact.

Figure 1f shows a variation of constant height STM where the tip apex is functionalized with a flexible hydrogen atom. The use of a flexible species at the apex of a metallic tip to provide enhanced contrast was first reported using CO-functionalized AFM tips to image the molecular structure of pentacene [28]. Other functionalisations of AFM tips have been explored including Cu–O tips [29–30] and Xe tips [31–32]. The use of H_2_ [33–34] and D_2_ [35] provided the first successful demonstration of the enhanced imaging contrast by scanning tunnelling hydrogen microscopy (STHM) [35]. Rather than direct tip functionalization as done in nc-AFM, STHM in these original studies was achieved by leaking in a background of molecular hydrogen (≈10^−9^ Torr) until an H_2_ molecule became trapped in the tip–sample junction. Here, we achieve STHM-like resolution by directly functionalizing the tip apex with a single hydrogen atom, picked up from the H–Si surface through the application of a voltage pulse, as reported in prior works [25–26,36]. Our ability to achieve STHM resolution using an H-functionalized tip aligns with recent STHM theory suggesting that the H_2_ molecule dissociates on the apex, resulting in a singly H-functionalized tip [37]. In Figure 1f, the use of STHM displays the surface as a series of squares with each of the square corners correlating to a H–Si atom. The image’s slight asymmetry can be attributed to a corresponding asymmetry in either the shape of the tip apex, or the attachment location of the functionalizing H atom. To highlight how differing asymmetries can affect STHM image appearance, Supporting Information File 1, Figure S3 shows a variety of images of the H–Si(100)-2 × 1 surface acquired with different H-functionalized tips.

A true measurement of the force interaction between the tip and sample can be visualized with frequency-shift maps generated by non-contact AFM [28]. In our work, we observe two different imaging modes that we denote as dark (Figure 1g) and bright contrast AFM (Figure 1h), based on the apparent contrast of the hydrogen atoms with the surrounding surface. Previous studies have identified the two contrasts as resulting from differing chemical reactivity of different functionalizing apex atoms [38], where the dark contrast image corresponds to a Si-terminated tip [39–41] and the bright contrast image corresponds to an H-terminated tip [39–40]. The transition between the two modes through the loss of H-functionalization of the tip (by capping a surface dangling bond [40]) can be seen in Supporting Information File 1, Figure S4. This differing chemical reactivity is seen in Figure 1j, where the height-dependent frequency shift spectra (Δ*f*(*z*)) taken above select positions on the surface (see Figure 1g,h for positions), shows significantly different character for each tip termination. To highlight the termination-dependent reactivity, frequency shift difference spectra [42] were calculated for both terminations (shown in the insets in Figure 1j). The spectra taken above an H–Si atom (green marker) and above a dimer bond (purple marker) were referenced to the “between dimer” position (blue marker). As evidenced by the differing inflection between the functionalization types, a stronger repulsive component is observed for the more inert H-functionalized tip probing the H-atom (bright contrast), while the more reactive Si-terminated tip (dark contrast) leads to a stronger attractive interaction (more negative Δ*f*) at the same site [39–41,43–45]. Simulations of the STHM, H-apex AFM, and Si-apex AFM images of the defect free H–Si(100)-2 × 1 surface using the probe particle model [23–24] can be found in Supporting Information File 1, Figures S5, S6, and S7, respectively.

Similar to how a change in tip functionalization can change the observed contrast (Supporting Information File 1, Figure S4), a fixed tip functionalization can exhibit inverting contrast if features of different chemical reactivity are scanned. Figure 1i,k shows the surface where the terminating hydrogen atoms were selectively desorbed from the upper half of the shown area. Examining the filled states STM image in Figure 1i, the bare silicon appears brighter when compared to its H-terminated counterpart [43]. Scanning the same area in AFM with an H-functionalized tip in Figure 1k reveals that the H-covered half (bottom) shows the rows as expected in bright contrast, but an inverted dark contrast for the unterminated portion (top). This demonstrates that the dark contrast is a result of the high reactivity between two different species – silicon and hydrogen – interacting with each other in the tip–sample junction, regardless of which species functionalizes the tip. The alternating asymmetry of the unterminated dimers along the dimer row is resultant from the buckling known to occur for the reconstructed bare surface [46–49], as well as a slight double tip artefact.

### Defect catalogue

**Figure 2:**
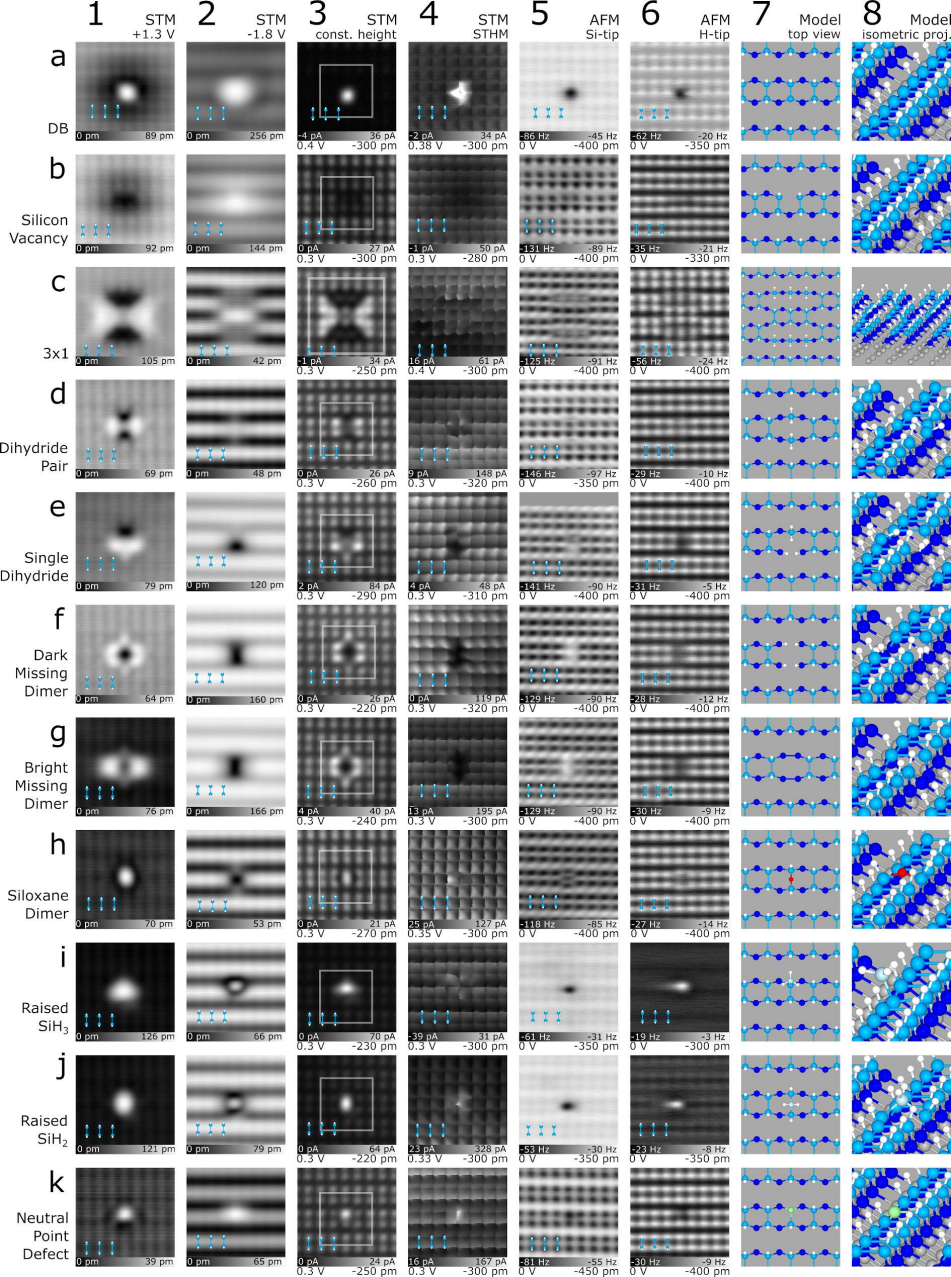
Common features of the H–Si(100)-2 × 1 surface imaged using the indicated STM and nc-AFM imaging modes. The identity of the features is listed on the left of the rows, with a ball and stick model of the dimer structure shown as an overlay in each scan (lower left corners, 0.77 nm width). The area of the structural models in column 7 is indicated by the grey box surrounding the defect in column 3. For the constant-height analysis types in columns 3–6, the applied constant bias value and relative tip–sample height are given in the lower left and lower right, respectively, of each frame. Colours in the ball and stick models are: H – white, Si dimers – royal blue, back-bonded Si – dark blue, O – red, raised Si – sky blue, and unknown species – green. Images are 3.2 nm × 3.2 nm.

Combining all six imaging modes allows for an in-depth characterization of the most common surface features and their local environments, as shown in Figure 2, arranged by their overall appearances. While not an exhaustive list, it features defects routinely seen when the surface is prepared using the procedure outlined in the Methods section. To categorize the defects one could focus on functional aspects (charged vs neutral features) [9], or structural aspects (including missing or additional atoms). Here, we categorize the defects on structural commonalities, based on the number of affected atoms. First, we list features that involve only one side of a dimer. These include a dangling bond (DB) of a surface Si atom (Figure 2a), a subsurface vacancy (Figure 2b), a SiH_3_ group (Figure 2i), and an unidentified surface point defect (Figure 2k). Second, defects affecting a whole dimer which include two missing H atoms creating a bare dimer (neutral, shown in [50–51]), two additional H atoms on a dimer to create a dihydride pair (Figure 2d), a single additional H atom on one side of a dimer to form a single dihydride which, in turn, requires the neighbouring Si atom to be missing (Figure 2e), the removal of the whole dimer (two missing Si atoms) accommodated by either H-termination (Figure 2f) or dimer formation in the second layer (Figure 2g). Alternatively, the dimer bond could be replaced by either a SiH_2_ group (Figure 2j) or an oxygen atom (siloxane bridge, Figure 2h), presenting as row-centred features. Remaining surface features are often a combination of two or more smaller structures, with one example being the often seen 3 × 1 reconstruction which is a combination of dimer rows and dihydride atoms (Figure 2c).

**Figure 3:**
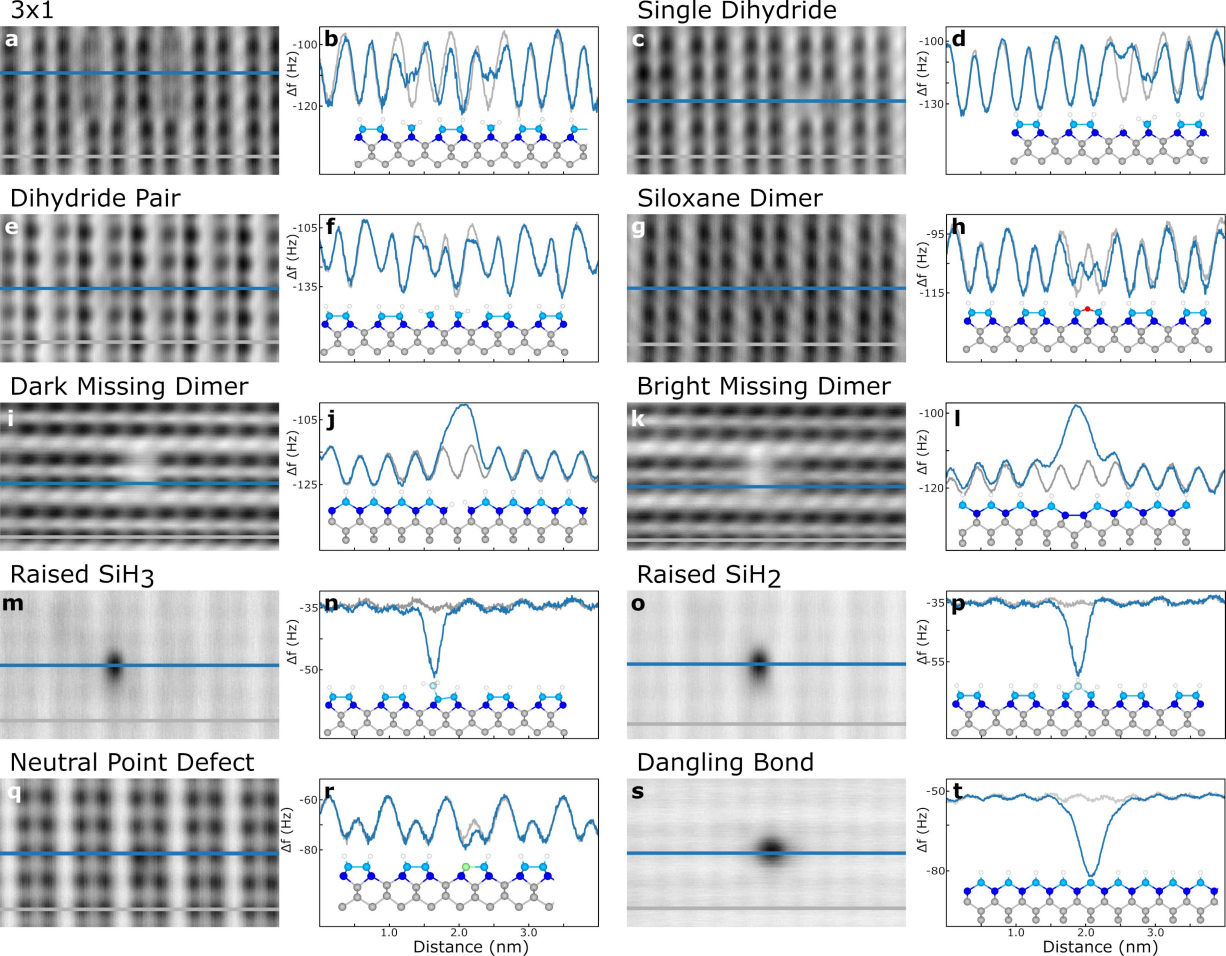
Analysis of defects using dark-contrast AFM and profile extractions. Line cuts of the AFM scans using Si-terminated tips were performed by averaging over 6 adjacent lines, with the thickness of the line profile indicating this averaging. The defect-including profiles are indicated in blue, while the defect-free profiles for each panel are in grey. The defect type is indicated above the relevant panels. See Figure 2 for the experimental image parameters.

**Figure 4:**
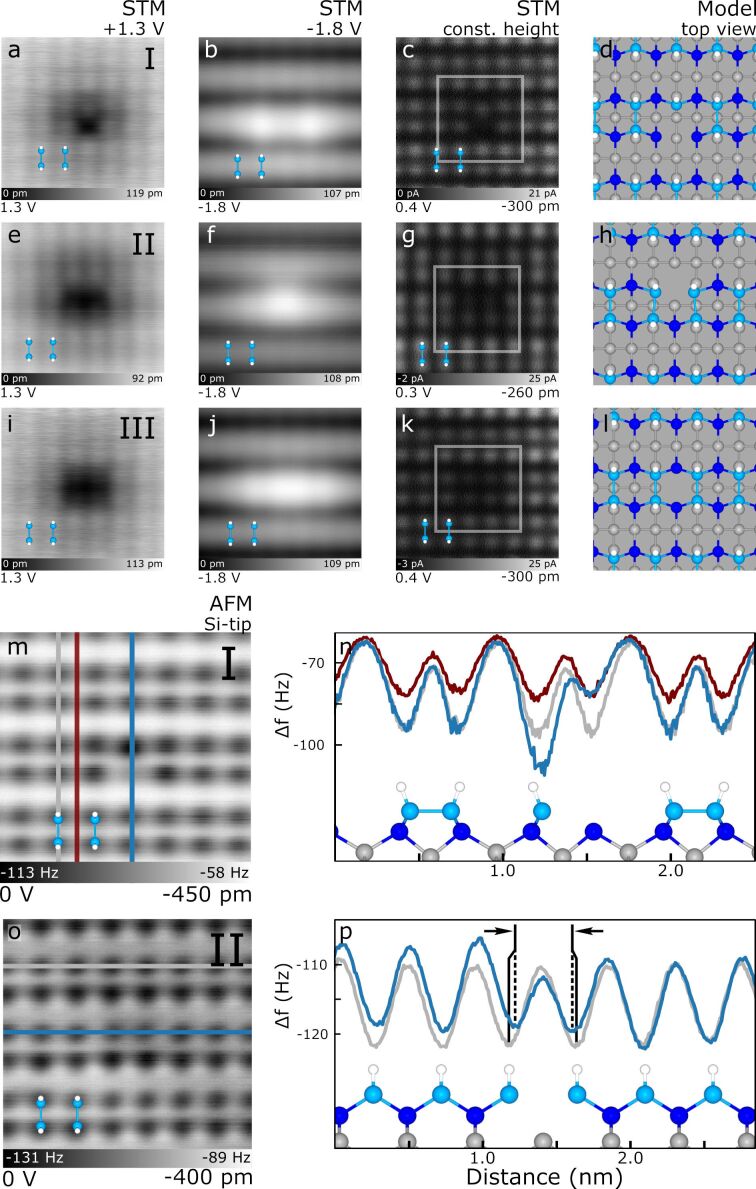
Silicon vacancies at different lattice sites. (a–d), (e–h), and (i–l) show the changing STM appearance of silicon vacancies located at different depths and lattice sites, labelled I, II, and III, respectively. A ball and stick model of the dimer structure is shown as an overlay in each scan (lower left corner, 0.38 nm width). Each STM image is 3.4 nm × 3.4 nm. (m,n) show the line cuts of vacancy I in (a) while (o,p) show the line cuts of vacancy II in (e) with the apparent change in atom separation highlighted (reduction of approximately 15%). Each AFM image is 2.8 nm × 2.8 nm. The defect-including profiles of n and p are indicated in blue, the defect-free profiles are in grey, and a background (between dimer pairs) profile is in burgundy. Colours in the ball and stick models are: H – white, Si dimers – royal blue, back-bonded Si – dark blue, and bulk Si – grey.

We start with a discussion of silicon DBs, which are well-studied unterminated silicon atoms [52–54]. In STM, the centre of a DB is imaged as a bright protrusion due to its conductive orbital which extends into vacuum. DBs have been observed to act like quantum dots and have discretized charge states in the bandgap of the material [6]. Due to the degenerate n-type doping of our substrate (see Methods) [6,53], DBs are natively negatively charged when imaging the empty states of the surface. This localized negative charge leads to band bending around the DB location at these biases, giving the DB a dark “halo” around the bright orbital protrusion (Figure 2a-1) [53,55]. Filled states imaging (Figure 2a-2) lacks the charge-induced band bending around the DB due to competing electron emptying and filling rates, rendering the DB neutral on average [54–55]. The constant height STM and STHM images in Figure 2a-3 and Figure 2a-4 also reveal the central protrusion. The STHM image showed an extremely localized current signal over the DB and has been artificially saturated from a 2.4 nA range to better show the H–Si atoms of the surface relative to the DB. A full-scale image of the DB is shown in Supporting Information File 1, Figure S8.

**Figure 5:**
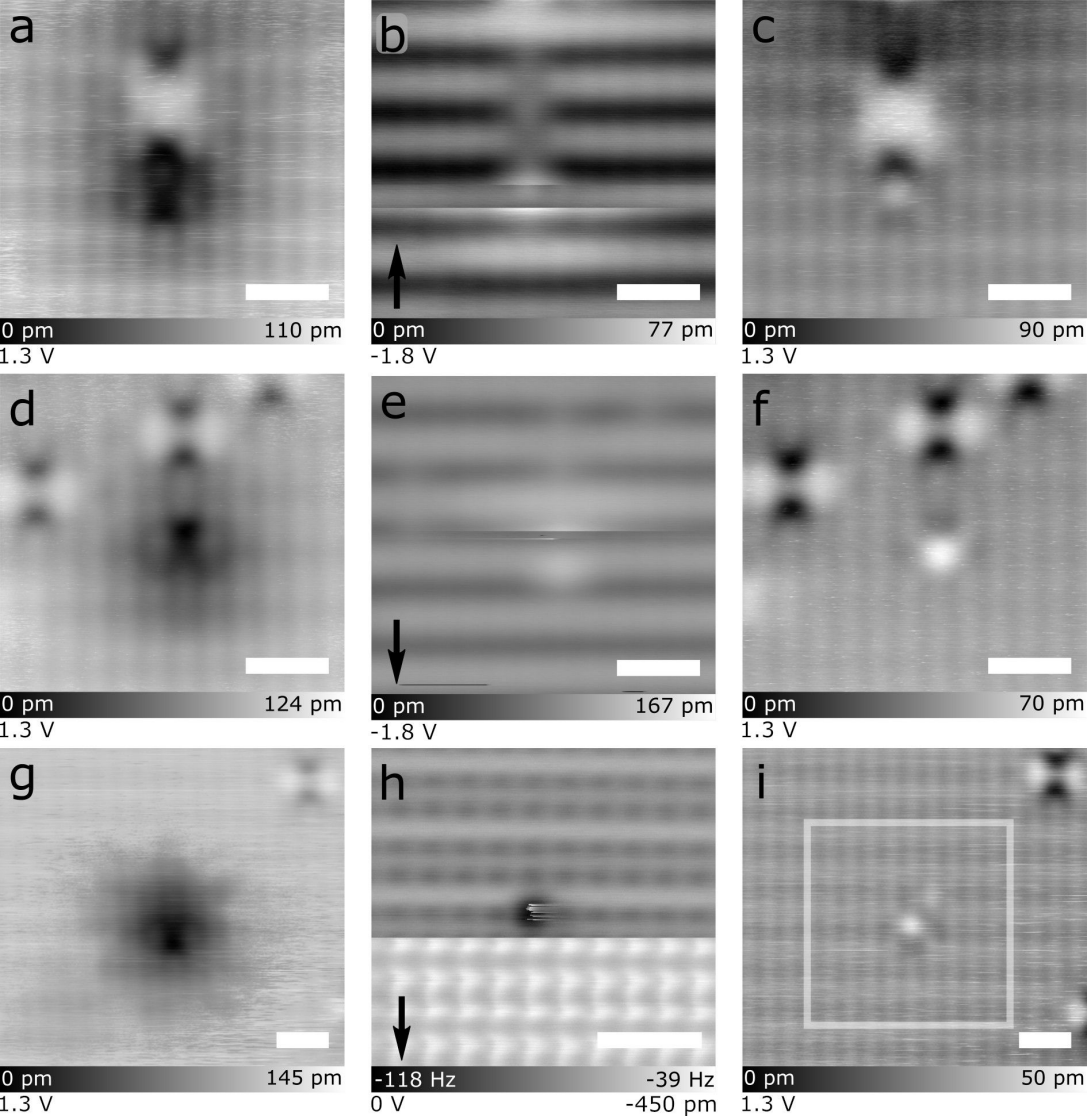
Tip-induced removal of hydrogen atoms decorating neutral point defects. (a–c), (d–f), and (g–i) show three instances of the apparent neutralization of a negatively charged defect. (a,d,g) Empty states images of the negatively charged H-decorated point defect before H liberation. (b,e) Show filled states STM removal events, while (h) shows an AFM removal event. The arrows in the lower left indicate the scan direction, with contrast changes in the scan indicating a removal event has occurred. (c,f,i) Empty states STM images of the same frames as (a,d,g), but after the H removal. The point defects no longer display charge-induced band bending around their location. The white box in (i) indicates the size of the scan frame in (h). Scale bars are 1 nm.

Finally, AFM analysis in Figure 2a-5 and Figure 2a-6 presents the DB as a large negative frequency shift, with both tip terminations seeing a strong attractive interaction (further details of all Si-tip based images in column 5 are discussed below with Figure 3). Since the Si-functionalized tip is predicted to be neutral [56–57], the dominant attractive contribution above the DB with both tip functionalisation’s is due to a covalent interaction between the surface DB and the H or Si atom of the tip [39,44–45]. Since the surface DB is negatively charged at 0 V, an additional attractive interaction due to the polarization of the tip is also expected to contribute to the strength of the attractive interaction [45]. To highlight the attractive contributions, Figure 1k from earlier can be referenced; the unterminated portion consists of many neutral bare dimers displaying as lighter in contrast than their single DB counterparts demonstrating the coulombic contributions (see the 4 dark DBs present at the edge between the bare and H-terminated surface) while both the bare dimer and single DB have an additional covalent interaction with the tip that is not seen above H–Si atoms. Complementary modelling of the DB defect and all subsequent defects of Figure 2 as imaged with STHM, H-apex AFM, and Si-apex AFM has been done using the probe particle model [23–24] and is discussed in the Supporting Information File 1, with Figures S5, S6, and S7.

Figure 2b shows a suspected silicon vacancy (discussed in more detail below in Figure 4), previously referred to as a type 2 (T2) defect in the literature [58]. Prior works speculated a variety of origins for this defect, including a negatively charged As dopant [58], Si-vacancy hydrogen complexes [9], and B dopants [59–60]. Crystal vacancies have previously been identified in other materials using scanning probe microscopy including Ga vacancies in GaAs [61], As vacancies in GaAs [62], and P vacancies in InP(110) [63]. Due to the common nature of these defects in other semiconductors and the similarity in imaging character, we thus attribute this defect to most likely be a Si vacancy. Further discussion of our assignment and additional evidence is given later as part of Figure 4. Empty states STM in Figure 2b-1 shows that the vacancy exists as a negatively charged species as evidenced by the reduction in brightness around the defect from charge-induced band bending. Unlike the DB, the vacancy’s charge state is observed to remain fixed even at filled states STM probing, with the charge-induced band bending enhancing the conductivity locally around its location, as seen in Figure 2b-2. In the constant-height STM frame in Figure 2b-3 a reduction of current due to the charge-induced band bending is again observed, along with a distortion of the density of states of the surface H–Si atoms above the sub-surface vacancy. 2b-5 and 2b-6 show similar distortions in the AFM frequency shift signal around the two H–Si atoms closest to the vacancy, suggesting a modification in their position or electronic character.

Next we discuss the raised SiH_3_ or silyl group (Figure 2i) [64]. This group exists where a silicon adatom replaces the H atom on one side of a dimer, achieving stability by saturation of the remaining free bonds with H. Larger groups of similar raised Si groups have been observed to bond and form islands [22,65]. The triangular shape of the defect as seen in the STM images (Figure 2i-1,2,3) highlights the preferred tetrahedral bonding orientation of the saturating hydrogens of the Si, with the fourth bond affixing it to the side of the dimer beneath. The characteristic dark border in Figure 2i-1 and enhanced brightness in Figure 2i-2 are features commonly observed in raised Si clusters of various sizes as discussed with Supporting Information File 1, Figure S9.

The raised nature of the silyl group has implications for all the performed constant height analysis; in constant height STM (Figure 2i-3) it dominates the current as it is closer to the tip, in STHM (Figure 2i-4) it leads to extended distortions due to the group’s high flexibility (only one bond affixing it to the surface), and finally it gives rise to stronger frequency shifts in nc-AFM (Figure 2i-5,6), again due to its closer proximity to the tip. The absence of the anticipated hydrogen splitting features of the SiH_3_ group is likely due to the added flexibility of the defect itself. Unfortunately, such an effect cannot be reproduced with the rigid structure used in the probe particle model as discussed with Supporting Information File 1, Figures S5, S6, and S7 and requires further theoretical verification.

Figure 2k shows a previously unreported neutral point defect. Experiments presented later as part of Figure 5, Figures S16, and S17 (Supporting Information File 1) show that this defect initially exists in a negatively charged, H-decorated state. It transitions to the neutral variety presented here by removal of the decorating hydrogen with the probe tip as the defect is scanned. This tip-induced H liberation occurs easily, rendering it difficult to obtain a full set of images of the H-decorated species. The neutral variation exists very close to the surface both spatially and electronically, as indicated by the height scales in the constant current STM images in Figure 2k-1,2. In the constant height STM image of Figure 2k-3, it shows only a slight increase in conductivity localized to a single atomic site, suggesting that the Si atom on one side of the dimer is perhaps replaced by this point defect. The AFM images of Figure 2k-5,6 confirm this localized nature, with the defect appearing as a slightly darker circular feature of enhanced reactivity. In addition to the absence of any charge-induced band bending surrounding the neutral point defect in empty states imaging, the defect was also shown to have no effect on the contact potential difference of the surface as measured with KPFM (Figure 1 of [9]).

We now move to a discussion of defects that affect a whole dimer, starting with dihydride pairs (Figure 2d) and single dihydrides (Figure 2e). Instead of a silicon bonding with its neighbouring Si atom to create a dimer, it can be saturated with 2 H atoms to create a dihydride. This leaves the second Si atom of the dimer to also bond with 2 H atoms (resulting in a pair of dihydrides (Figure 2d)) [21,66–69] or be absent (resulting in the single dihydride of Figure 2e). The concentration of dihydrides can be controlled by lowering the annealing temperature during sample preparation [19,70]. While the two varieties of dihydride look unique overall in STM empty states topography (Figure 2d-1 and Figure 2e-1), the side of the pair that the dihydride unit(s) appear on consistently presents as a dark depression. A reversed trend is seen in filled states imaging (Figure 2d-2 and Figure 2e-2), with the dihydride side(s) diffusely bright. In constant height STM imaging (Figure 2d-3 and Figure 2e-3), dihydride sites image as areas of reduced current, with neighbouring dimers showing an enhanced current [64]. The STHM image of Figure 2e-4 provides an interesting example of STHM’s utility, allowing for a direct comparison between the dihydride and a missing atom. At the dihydride site (top of row) we observe a significant deviation from the square pattern, suggesting that additional atoms are present. The missing atom portion of Figure 2e-4 (bottom of row) conversely shows darkening and an absence of the expected right-angled intersections that make up the corners of the squares, suggesting a missing feature. Finally, dihydrides provide a good example of the benefit of different AFM imaging contrasts. Examining the dark contrast AFM images based on Si-terminated tips in Figure 2d-5 and Figure 2e-5, the dihydride(s) possess a dark splitting feature, corresponding to the presence of the two H atoms. The difference in contrast of the splitting feature between the two dihydride variations is believed to originate from a tilting of the hydrogen atoms in the dihydride pair relative to the surface plane and will be discussed in more detail with Figure 3. Contrarily, the splitting of the dihydride site is not readily apparent in the H-functionalized AFM images (Figure 2d-6 and Figure 2e-6), presumably due to the flexibility of the H-sensitized tip or its lower reactivity. The dihydride pair in Figure 2d-6 becomes almost indistinguishable from the background and the missing silicon site as part of the single-dihydride in Figure 2e-6, however, does show up as an area of reduced frequency shift.

Next, we discuss two variations of missing dimer defects denoted as dark missing dimer (Figure 2f) and bright missing dimer (Figure 2g) [14,71–72]. These missing dimer defects appear almost identical for most analyses done, except when comparing the STM empty state images in Figure 2f-1 (dark) and Figure 2g-1 (bright). To support that these two defects are unique and not the consequence of a tip change, the data were taken sequentially while continuously monitoring for tip changes (except STHM), ensuring identical apex character. The two varieties originate from different reconstructions of the exposed next-layer silicon atoms, similar to what was found on the unterminated surface [73]. The formation of two dimer bonds – orthogonal compared to the top layer dimers (model in Figure 2g-7,8) –corresponds to the bright variation (Figure 2g), and H-termination of all exposed sites in the second layer (model in Figure 2f-7,8) leads to the dark variation (Figure 2f). The latter has been reported to cause less lattice strain [74], matching our observation of less distortions in the STHM and nc-AFM images near the missing dimer (Figure 2f-4,5,6 compared to Figure 2g-4,5,6 and shown in Figure 3). The single dihydride defect of Figure 2e has also been observed to show bright and dark variations surrounding the missing Si atom shown in Supporting Information File 1, Figure S10.

Figure 2c explores the 3 × 1 reconstruction of H–Si [20,75–76]; a structure composed of a combination of H-terminated dimers and dihydrides. Instead of two parallel dimer rows as in the normal surface reconstruction, there exists only a central dimer row of variable length (here we show one that is 3 dimers wide), with the remaining unpaired silicon atoms fully saturated as dihydrides (model in Figure 2c-7,8). STM imaging in Figure 2c-1 and Figure 2c-3 show an enhancement in conductivity at dimers neighbouring the reconstruction and a reduction above the H_2_Si atoms. Figure 2c-2 shows a dimer row that has been formed between two of the regular dimer rows, with Figure 2c-3 also highlighting this realignment. The dihydrides in the 3 × 1 reconstruction show similar features to the single and paired dihydrides discussed earlier, including the distortions in the STHM image from the presence of H_2_Si (Figure 2c-4) and the split-appearance of the two hydrogens in the Si-tip AFM scan (Figure 2c-5). A direct comparison of the differences observed in nc-AFM using a Si-tip (dark mode imaging), including the assignment of individual H atoms at the dihydride sites, is given later in Figure 3.

Defects that we observe to be centred within a dimer manifest themselves through the insertion of additional atoms between the dimer bond. We show a SiH_2_ group (Figure 2j) and an oxygen atom (Figure 2h) in such a position. Starting with the SiH_2_ group, it represents a silicon adatom in the bridge position between two top layer silicon atoms (model in Figure 2j-7,8). It can be compared to the earlier discussed SiH_3_ group, with both groups showing similar image contrast in STM topography (compare Figure 2i-1,2,3 with Figure 2j-1,2,3 and Supporting Information File 1, Figure S9). Their distinct location with respect to the dimer, however, is easily discerned throughout all analysis types in Figure 2i,j. In Si-tip AFM images (Figure 2i-5 and Figure 2j-5), the chemically reactive silicon tip strongly interacts with both defects. Conversely, images with H-functionalized AFM in Figure 2i-6 and Figure 2j-6 shows a less reactive character, with the inert tip probing inert hydrogen-saturated groups. At close defect-tip separation they should mutually repel each other resulting in a less negative frequency shift over the group location, as shown. Again, AFM does not allow one to discern among individual hydrogen atoms due to the assumed flexible nature of the raised groups, creating a blurred appearance in AFM imaging as the group is pushed during the raster scan.

The siloxane dimer of Figure 2h, previously denoted in the literature as a split dimer [77–78] and also incorrectly identified as a dihydride [2,79], is thought to be an oxygen bonded between the two Si atoms of a dimer. STM imaging reveals a localized defect with only subtle impact on neighbouring dimers (Figure 2h-1,2,3). STHM in Figure 2h-4 shows slight variation from the regular box-like appearance of the 2 × 1 dimer, with the positions of the atoms in the oxygen-bridged dimer further apart and a central bright feature present. This agrees with the Si-tip AFM data in Figure 2h-5, showing that the “dimer” indeed contains a third atom in the centre. In bright contrast AFM, the attractive interaction between the H-tip and the oxygen’s non-bonding electron pair leads to a slight depression in the centre, with the dimer’s H atoms “pushed out” (Figure 2h-6). A defect of similar appearance was reported in a prior work exploring chlorine-terminated silicon [80] which was linked to water contamination in the vacuum chamber (observed as H and OH bonded to the unterminated surface [15–17]). A mild annealing followed by halogen-termination allowed the oxygen to enter into the dimer, creating an Si–O–Si bond [81]. The authors commented that they expect this feature to also be present on hydrogen-terminated silicon, which we support here with our analysis.

This family of defects explored in Figure 2 underlines the importance of combining several modes of SPM imaging to differentiate among structures that might otherwise be assumed equivalent. Each imaging mode highlights different defect features so that when combined, a greater understanding of the defect is achieved. In general, however, we found AFM with Si-terminated tips to often be the most discriminating. It consistently demonstrates the highest spatial resolution, presumably due to the Si-terminated apex’s greater chemical reactivity and reduced apex flexibility. Thus, we employ Si-tip based AFM imaging to further examine select defects.

### Details of nc-AFM using silicon-terminated tips

In Figure 3, we compare AFM line profiles taken across the defect of interest (blue) and a corresponding defect-free region (grey) with a simple theoretical structure optimized by molecular dynamics calculations (see Methods for parameters). A preliminary comparison of Figure 3 with the simulated line profiles is shown in Supporting Information File 1, Figure S11. We start by comparing dihydride-based defects. Figure 3a shows a Si tip AFM image of a 3 × 1 reconstructed region, with the locations of the two comparative line profiles marked. Figure 3b plots the extracted cross-sections (averaged over the thickness of the lines), which are matched to the proposed structure at the bottom of the panel. This analysis highlights the dimer row that is out of phase with the surrounding dimers and the earlier discussed splitting feature observed over the two dihydride-hosting atoms. Similarly, a single dihydride adjacent to a missing atom is displayed in Figure 3c,d, with the dihydride again showing a splitting feature. The slight depression at the location of the missing Si atom (blue curve displays less frequency shift compared to the defect-free grey curve) suggests that the tip cannot fully probe the vacancy’s depth. Both of these dihydride variants can be compared to the final example of a dihydride pair in Figure 3e,f. It interestingly appears remarkably similar to the normal dimer cross-section, with only slight variation at the position of the outermost H atoms. The lack of a hydrogen-related splitting feature, as seen with the other dihydride species, can be explained by looking at the modelled geometry of the two closely-spaced dihydride species in the dimer in Figure 3f. Local repulsion between the two nearest hydrogen atoms on the inside of the pair results in the tilting of the dihydrides with respect to the surface. Thus, the position of the outer two hydrogens are now further away from each other and closer to the underlying bulk atoms, making the feature less visible to the AFM tip.

Examining the siloxane dimer in Figure 3g,h, three distinct minima are in place of the dimer. As mentioned earlier, the outer two hydrogen atoms spatially shift to accommodate the oxygen atom, extending the dimer structure by approximately 30% compared to a regular dimer.

Particularly interesting is the comparison between the two variations of the missing dimer. Figure 3i,j probe the dark missing dimer in which the unsatisfied bonds of the back bonded Si atoms are terminated with hydrogen. As such, any lattice strain is reduced and the neighbouring dimers show minimal variation compared to the defect free profile. Conversely, the maxima of the bright missing dimer in Figure 3k,l is accompanied by two local minima “shoulders”. These have a less negative frequency shift, with the minima pulled spatially towards the centre of the defect. We propose that this observation is a result of the second layer Si atoms forming dimer bonds at the defect location, introducing horizontal lattice strain. This pulls the neighbouring atoms inwards more than in the dark missing dimer case, accounting for the shoulders in 3l which are absent in 3j. Importantly, these two defects were imaged in succession, ensuring any variations are a consequence of their differing nature, not a tip alteration. The slight asymmetry between the profiles on the left and right of the missing dimers in both cases is a result of a slight tip asymmetry.

The two raised Si species in Figure 3m,n (SiH_3_) and Figure 3o,p (SiH_2_) present an extra challenge to analyse, as they must be imaged with a larger tip–sample separation to prevent damaging tip contact. As a result, the magnitude of the frequency shift of the unperturbed surface is small compared to the strong defect signal. Despite this, examination of the cross-sections for the SiH_3_ and SiH_2_ respectively, resolves the position of the defect with respect to the lattice; the SiH_3_ originates above one side of a dimer, while the SiH_2_ is centred between the two atoms of a dimer.

The neutral point defect in Figure 3q,r displays as a slight decrease in the minima above the defect. The almost normal appearance of the defect cross section compared to the regular surface suggests a similarly coordinated substitutional species. A slightly more negative frequency shift is observed suggesting the defect is chemically different from the surrounding H–Si atoms. As discussed with the simulations in Supporting Information File 1, Figures S5, S6, and S7, such a feature is not captured with the probe particle model suggesting that additional theoretical work is needed to identify its true nature.

Lastly, a lone DB shows up as a localized feature of enhanced reactivity when compared to the surrounding H-terminated surface (Figure 3s,t). This reactivity extends spatially away from the DB location, generating a broad minimum that eclipses the signal from neighbouring lattice sites. Due to the DB’s highly reactive nature, AFM imaging of DBs must be done with significant tip–sample separation to prevent any alteration to the tip or surface.

### The silicon vacancy

We now focus on an in-depth analysis of the silicon vacancy defect introduced in Figure 2b. While all of the other defects examined are observed with the same consistent appearance, there are several distinct species we attribute to the proposed Si vacancy. Figure 4 shows empty states, filled states, and constant current images of 3 different configurations of the defect, corresponding to different vacancy depths. Each of the configurations in Figure 4a–d, e–h, and i–l is denoted in the provided ball and stick models. These are all predicted to exist as negative charge centres, owing to the presence of unsatisfied bonds of atoms neighbouring the vacancy, which localize charge due to the degenerate doping of the crystal (see Methods for sample details). Starting with Figure 4a (labelled I), the defect is centred around a surface lattice site affecting one side of a dimer, with the negative charge bending the bands down locally as evidenced by the radial dark depression around the defect centre. The filled states STM image of 4b shows an increase in measured height, correlating again to the fixed negative charge of the defect [58]. Looking at the constant height STM image in Figure 4c, it appears that no atom is present at the defect lattice site. This is supported through examination of the AFM line cuts of this defect in Figure 4m,n (and Supporting Information File 1, Figure S12), which show the expected missing atom as an area of less negative frequency shift (blue curve in Figure 4n). Due to a missing first-layer silicon atom, the AFM probe is actually measuring a signal from the back-bonded and bulk silicon atoms. This is verified by comparing the magnitude of this smaller frequency shift to an equivalent measurement of other back-bonded Si atoms, as would be measured in a cross section taken between dimers (burgundy line in Figure 4n). Their similar magnitude supports there is no atom present, while also lending evidence to its correct classification as a Si vacancy [82–83]. Additionally, a more negative frequency shift minimum is seen in Figure 4m,n above the remaining atom of the dimer, with a shift of the minimum toward the site of the vacancy as shown in the blue cross-section in Figure 4n. The localization of the subsurface vacancy’s charge through the unsatisfied DBs is thought to prevent the otherwise required dihydride (Figure 2e) at the site of the neighbouring atoms from forming although further theoretical exploration is required to support this. A potential variation of the vacancy where one of the backbonded DBs is terminated with an H is presented in Supporting Information File 1, Figure S13 and the probe particle modelling of the Si vacancy “I” variety shown in Figure S14.

Figure 4e–h shows another variant of the silicon vacancy (labelled II). The broad features of the STM probing vacancy II in Figure 4e–g are similar to vacancy I, however, it can be seen that the defect no longer appears to affect a single atomic site, but rather reduces the apparent height of two adjacent dimers as shown in Figure 4g. While the subsurface defect cannot be directly probed, the similarities it shares with Figure 4a supports its assignment as a Si vacancy below the surface (see the ball and stick model in Figure 4h,p). This is further corroborated by line-cut analysis of vacancy II in Figure 4o,p. The two atoms centred above the defect in 4t show a reduced minimum, as well as a horizontal shift in position towards the defect centre due to a polaronic distortion induced by the vacancy’s localized negative charge. Molecular dynamics relaxation was unable to capture this effect as part of the modelling, so the ball and stick models of Figure 4h,p have been manually edited to show this effect.

Figure 4i–l shows the third common type of Si vacancy (labelled III). This defect shares similar STM features when compared to the previous vacancy examples, but is spatially shifted with respect to the Si lattice. In detail, the dark depression in Figure 4i is now symmetric in appearance and centred in the dimer row, the bright enhancement in Figure 4j extends over many dimer pairs, and in the constant height STM in Figure 4k the two dimers above the defect centre show a reduction in apparent height. Aligning the centre of this defect to a model of the underlying Si lattice (see Figure 4l) and factoring in that the missing atom must preserve the observed experimental symmetry, it is likely the position of this vacancy is in the third atomic layer (missing grey atom in the centre of Figure 4l). While our assignment of this defect as a Si vacancy is consistent with our experimental results, detailed modelling will be needed to investigate the various rebonded and H-capped alternatives and compare them to the STM and AFM images.

### The neutral point defect

We now return to a discussion of the neutral point defect, which we noted initially exists in a negatively charged, H-decorated state. Figure 5 provides three examples of tip-induced transitions of a negative species to the neutral point structure. For each case, the leftmost panel shows a dark depression scanned at positive STM bias that is associated with a localized negative charge (similar to the dark halo around a DB in Figure 2a-1 or the vacancies in Figure 4). At this point, one could be tempted to assign the dark feature to the same vacancy defect as in Figure 4 due to their similar likeness. However, this species is found to be unstable and irreversibly altered upon scanning at negative bias (in constant current mode) or zero bias (with reduced tip–sample separation in AFM mode) (Figure 5b,e,h), with the consistent observation of a sharp discontinuity close to the site of the defect. The discontinuity is accompanied by a change in appearance of the defect which remains in the subsequent empty states images (compare Figure 5c,f,i to the first scans in Figure 5a,d,g). Importantly, we observe the complete absence of a charged species after the transformation and a replacement with the neutral point defect. The change in contrast of the AFM image of Figure 5h further suggests that the tip apex transitioned from a Si-tip to a H-tip through the liberation of the H atom decorating the defect. Efforts to replace an H atom on such a site were unsuccessful.

Furthermore, our assignment that the neutral point defect acts as a hydrogen trap during sample preparation is based on the similarity of the defect’s negative state to physisorbed hydrogen atoms on the surface (see Supporting Information File 1, Figure S15 for STM images of physisorbed hydrogen atoms). Prior work reported that lone hydrogen atoms are negative on a H-terminated degenerately doped n-type sample [9], while another reported they could be picked up from the surface during filled-states STM imaging [26]. Additional examples of the H removal along with STM *I*(V) and d*I*/d*V* spectroscopy of the neutral point defect can be seen in Supporting Information File 1, Figures S16 and S17. This evidence, together with the fact that our sample preparation methodology produces many hydrogen radicals that can penetrate the surface, supports the idea that the neutral point defect behaves as a hydrogen trap. As to its identity, it has been reported in the literature that boron, when added to silicon, can behave as a hydrogen trap [84–87]. Its trivalent nature would place the atom in a neutral charge configuration when substituted for a Si atom within a dimer. The addition of a hydrogen atom could then force a weakly held bond to form, allowing for the localization of an additional electron leaving the boron in a negative charge state. While this seems to support its assignment as boron, the areal concentration these neutral point defects are observed at (0.1–7.6 defects/10 nm^2^) is higher than would be expected for contaminant boron from commercial wafer processing [58]. Further investigation into the origin of this defect will be required for a conclusive determination.

## Conclusion

In this work we have created a comprehensive catalogue of commonly found defects on the H–Si(100)-2 × 1 surface analysed using a combination of several STM and nc-AFM imaging modes, with reproducibly formed tip terminations of different reactivity. Through this analysis we are able to identify unique electronic and structural signatures associated with the defects of each imaging mode. By combining these results with a simple probe particle simulation of the STHM and AFM images, we were able to confirm the underlying mechanism of STHM, the classification of several surface defects, as well as more confidently identify the previously reported T2 defect as having character more consistent with a silicon vacancy. Finally, we examined the previously unreported neutral point defect, observing its transition from a negatively charged species by means of tip-induced liberation of an atomic hydrogen from the defect site. While our work presents a comprehensive understanding of the experimental nature of these defects, additional theoretical studies using more powerful techniques such as DFT is still needed to confirm the results presented here and provide a potential classification of the point defect. We also note that it is expected that other sample termination methods, Si wafers, and vacuum systems could potentially lead to additional defects not reported here. While this means this is not an exhaustive list, our analysis provides fresh insight into the nature of many commonly observed defects. Through this now enhanced understanding of the nature of the most common defects, we enable informed refinement of standard H–Si wafer preparation methods, leading to a more reliable platform for the creation of these devices.

## Methods

Experiments were performed using an Omicron LT STM and an Omicron qPlus LT AFM [88–89] system operating at 4.5 K and ultrahigh vacuum (3 × 10^−11^ Torr). STM tips were electrochemically etched from polycrystalline tungsten wire, resistively heated, and field-evaporated to clean and sharpen the apex using a field ion microscope (FIM) [90]. AFM tips used the third-generation Giessibl tuning forks with a FIB mounted tungsten tip (*f*_0_ ≈ 28 kHz, *Q*-factor ≈ 16k–22k) [91]. The tip was cleaned and sharpened in vacuum using a FIM [90]. In situ tip conditioning was done by executing controlled contact on a hydrogen-desorbed patch of silicon [43,92]. Bright contrast, H-functionalised AFM tips were routinely formed with controlled contact on H-terminated portions of the surface, while bare silicon AFM tips were formed using only desorbed patches (although not all controlled contacts on a desorbed patch returned a Si-tip). STHM tips were achieved by creating DBs via tip pulsing, where the desorbed H is reported to sometimes decorate the tip’s apex [25–26,36], providing contrast as shown in Supporting Information File 1, Figure S1a.

The samples used were highly arsenic-doped (≈1.5 × 10^19^ atoms/cm^3^) Si(100). Samples were degassed at 600 °C overnight followed by multiple cycles of flash annealing at 1250 °C. The samples were then terminated with hydrogen by exposing them to molecular hydrogen (10^−6^ Torr) while the Si sample was held at 330 °C for 2 min. The molecular hydrogen was cracked from H_2_ gas using a tungsten filament held at 1600 °C [93].

Image and data acquisition was done using a Nanonis SPM controller and software, with the imaging parameters for each of the 6 SPM analysis modes described in the text. The height setpoint reference was taken as the tip–sample separation over a H–Si atom with an imaging bias of −1.8 V and a current setpoint of 50 pA. The exact magnitude of the Δ*f*(*z*) spectroscopy changed between tip shaping events, but the general shape and behaviour for H- and Si-terminated tips (bright and dark contrast) remained consistent throughout multiple tips and tip terminations.

The defect free H–Si ball and stick model was the same as used in [43], with defects manually inserted using Avogadro [94–95]. The geometry of the defect atoms within the lattice were optimized using molecular dynamics relaxation with a Merck molecular force field (MMFF94) [96]. Images of the lattice were colourised and rendered using Mercury [97]. Details of the probe particle simulations can be found in Supporting Information File 1.

## Supporting Information

File 1 Referenced images and a supporting discussion of the probe particle simulations.

## Data Availability

Data is available upon request from the corresponding author.
