# Supplementary material for: Atomic defect classification of the H–Si(100) surface through multi-mode scanning probe microscopy
Source: Beilstein J Nanotechnol. 2020 Sep 7;11:1346–60. doi: 10.3762/bjnano.11.119 (PMC7492692; doi:10.3762/bjnano.11.119)
Supplement: File 1 — Referenced images and a supporting discussion of the probe particle simulations. [file Beilstein_J_Nanotechnol-11-1346-s001.pdf]

BEILSTEIN JOURNAL OF NANOTECHNOLOGY

Supporting Information

for

Atomic defect classification of the H–Si(100) surface through multi-mode scanning probe microscopy

Jeremiah Croshaw, Thomas Dienel, Taleana Huff and Robert Wolkow

Beilstein J. Nanotechnol. **2020,** *11,* 1346–1360. doi:10.3762/bjnano.11.119

Referenced images and a supporting discussion of the probe particle simulations

License and Terms: This is a supporting information file under the terms of the Creative Commons Attribution License (https://creativecommons.org/licenses/by/4.0). Please note that the reuse, redistribution and reproduction in particular requires that the authors and source are credited.
The license is subject to the *Beilstein Journal of Nanotechnology* terms and conditions: (https://www.beilstein-journals.org/bjnano)

Table of Contents

- Additional figures of defects at various imaging conditions, tip sample separation, and tip functionalization.
- Probe Particle Simulations
    - Description and method
    - Figures with direct comparison between experiment and simulation
    - Discussion of results and limitations of simulation approach
- Additional figures
- Hydrogen removal from point defects
    - High resolution imaging of actual event and spectroscopy
    - Sequence of large area scans showing multiple events

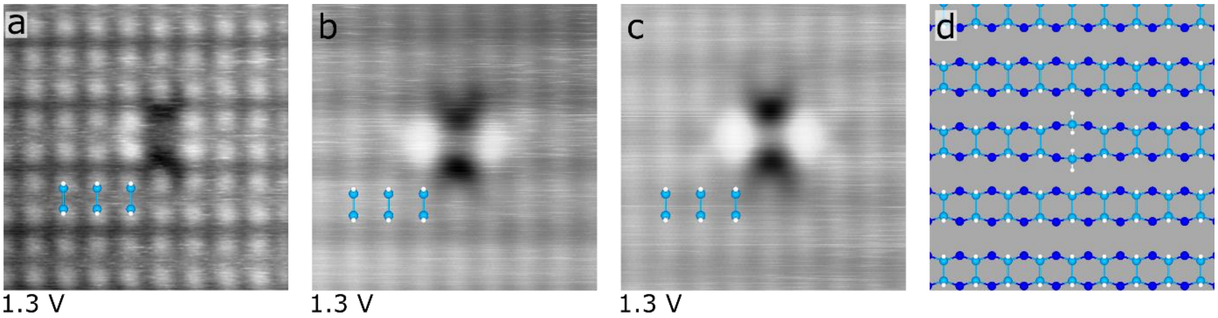

Figure S1. Variation in dimer contrast in constant current STM topography of empty states imaging (1.3 V, 50 pA). A dihydride pair aids to identify the dimer rows in each case. (a) Single atom contrast, where each H-Si atom is readily assignable. (b) True dimer contrast, aligned with the dihydride pair. (c) False dimer contrast where apparent dimer features appear offset from the dihydride pair. (d) The corresponding ball and stick structure for a dihydride pair. The high-resolution contrast in (a) is routinely achieved by functionalizing a dark contrast Si-terminated tip with a single hydrogen atom through the creation of a DB (H-functionalization, the reverse process is discussed in Figure S4). This H-functionalized tip then shows bright contrast and can be used for STHM. No correlation has been observed for the AFM contrasts and the STM dimer contrasts in (b) or (c). Each image is 3.4 x 3.4 nm^2.

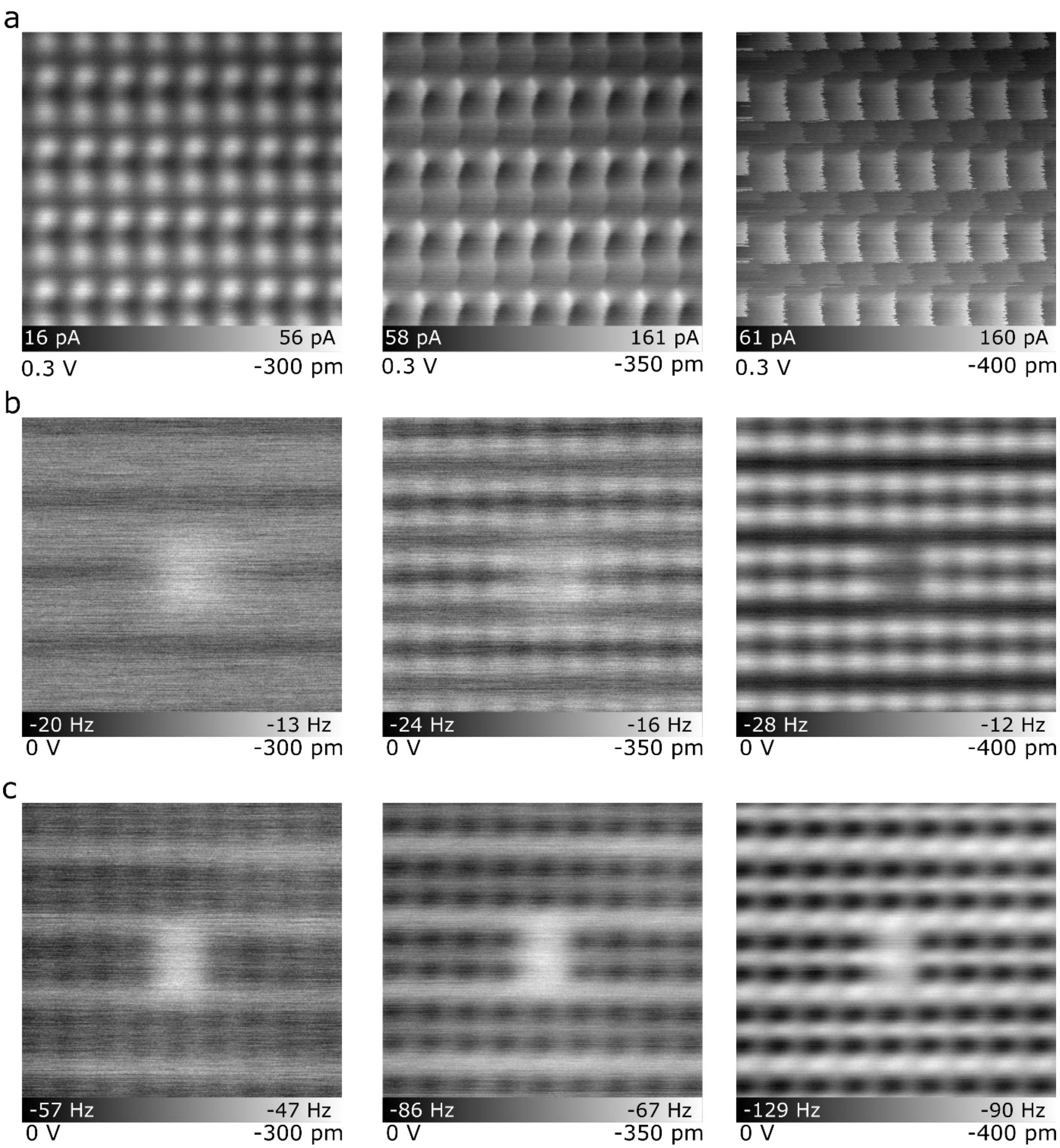

Figure S2. Height Series of Constant Height Imaging. (a) Constant height series of defect free H-Si(100)-2x1 with an STHM tip. (b) Constant height Δf series of a dark missing dimer with a H-terminated AFM tip. (c) Constant height Δf series of a dark missing dimer with a Si-terminated AFM tip. Each image is 3x3 nm^2 with imaging parameters indicated below each image.

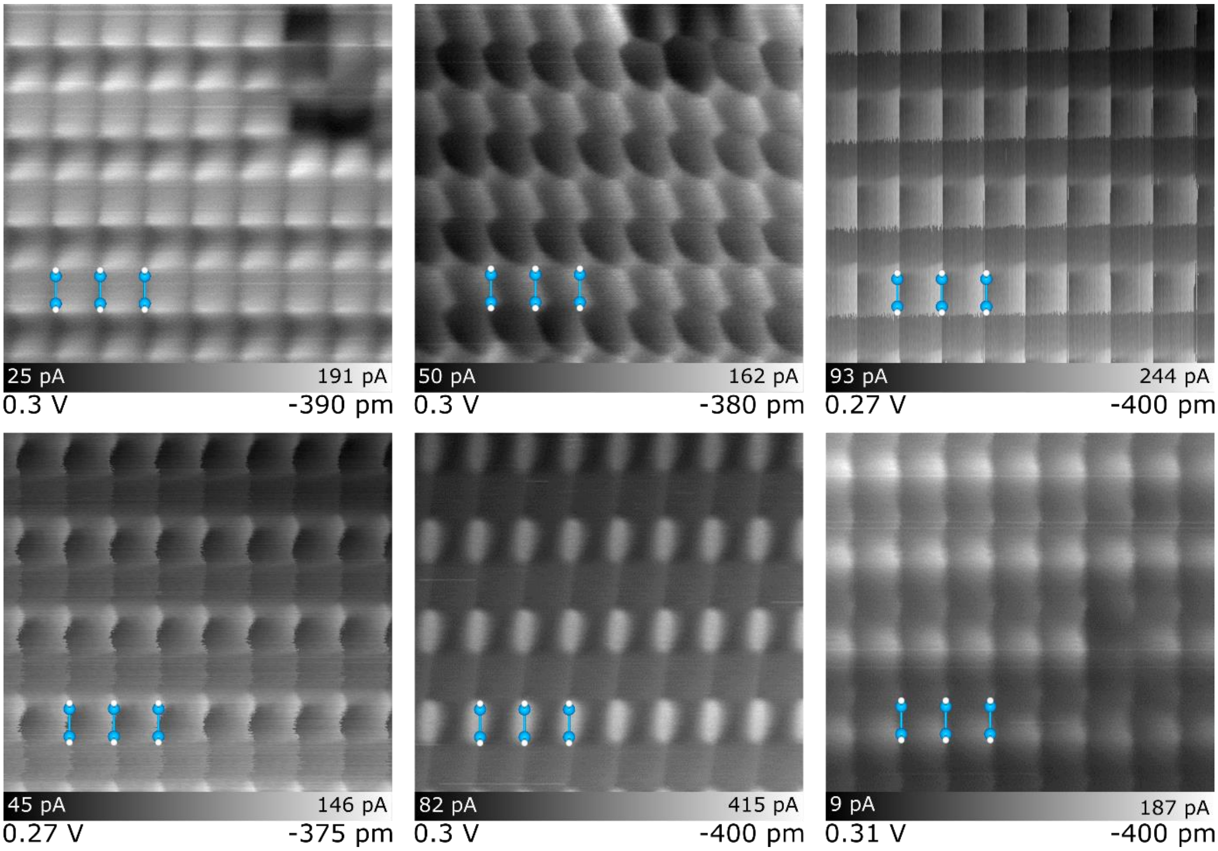

Figure S3. STHM Images of the H-Si surface taken with different H-functionalized tips of differing flexibility. The variation in surface character between the images highlights how asymmetries of the tip, or variations in apex coordination can affect the observed imaging. Each scan is 3.1 nm x 3.1 nm.

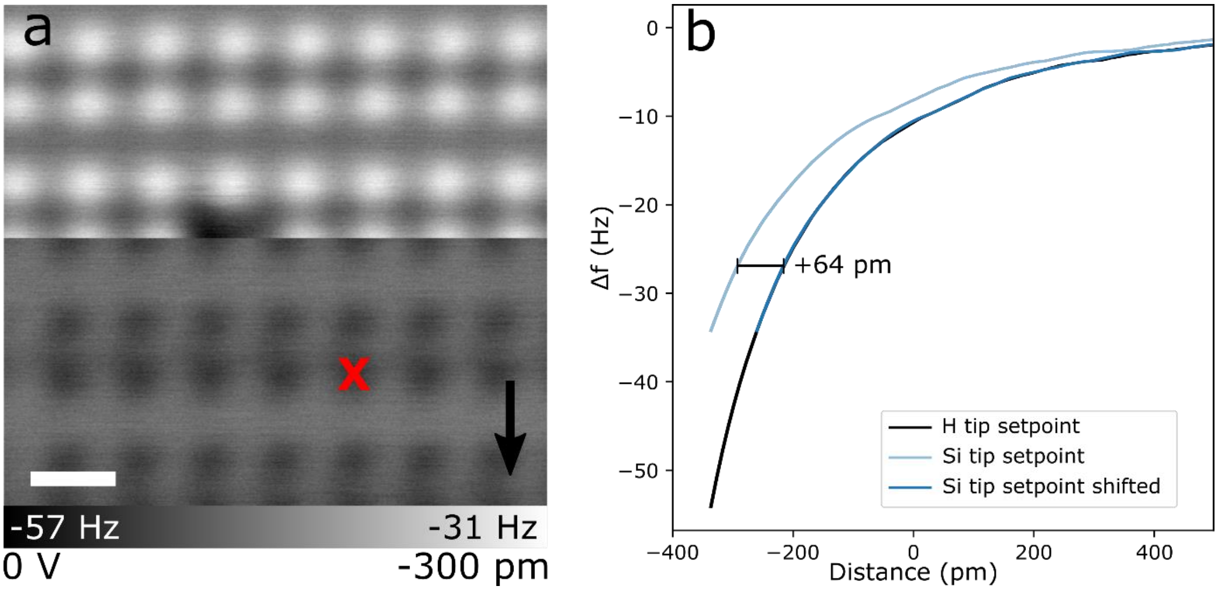

Figure S4. Relative height difference between a Si and H apex tip. (a) Δf map with a contrast change observed mid-scan from changing apex functionalization. An originally dark contrast silicon tip was functionalized with a hydrogen atom through the creation of a DB so that it exhibits bright contrast (top half of image). That H-functionalized tip was then scanned over a DB resulting in a H transfer from the tip to the sample and a corresponding change in contrast (silicon terminated tip for bottom half of image). (b) A Δf(z) spectroscopy was taken over a H-Si atom after the capping event (red X in (a)) and once more after resetting the height setpoint with the silicon-terminated tip (dark contrast). By comparing the curves, it is observed that the H-terminated tip sits 64 pm closer to the surface. While this height difference is roughly 2x smaller than a H-Si bond, it is important to note that this measured height is a convolution of the H-Si bond and the difference in height setpoint as a result in different density of states between the Si-terminated and H-terminated tip. The arrow in (a) indicates the scan direction, the scale bar in (a) is 500 pm. The height in (a) is the distance from a setpoint of -1.8 V and 50 pA with a H-functionalized apex. The spectroscopy curves were smoothed using a Savitzky-Golay filter to make it easier to align the plots.

Probe Particle Simulations

In addition to the experimental results, simulations based on the probe particle model were used to reproduce the STHM and AFM images of the observed defects. The probe particle model developed by Hapala *et al.* works by simulating a probe particle which is attached to a metal tip and allowed to distort based on interactions with the surface. These interactions are described using empirical potentials following a pairwise Leonard-Jones potential which maps the interaction between the probe particle and the surface, and the probe particle and the metallic tip to which it is held[1]. The structure of the sample surface used in the probe particle model is generated by starting from a defect free H-Si slab from Ref. [2], adding the defects and relaxing to their respective geometry using a molecular dynamics package (Merck Molecular force field

MMFF94)[3]. The resulting structures are shown in ball and stick representation throughout the paper.

The imaging parameters for the simulations were kept very close to those available in the provided examples[4]. Simulations done to mimic the STHM images were performed with a flexible H atom at a tip sample separation of roughly 780 pm defined as the distance between the surface's Si atoms and the metal apex of the tip. All H-apex AFM images were simulated using the same tip conditions as the STHM images except the tip sample separation was increased to roughly 870 pm. The Si-apex AFM images were simulated at the same depths as the STHM images except the apex atom was replaced with a Si atom and the tip spring constant, which affects the probe particle flexibility, was increased by 40 times.

As we will discuss in the following, the simulated images show several significant successes and shortfalls in resembling the features of the experimental images. The STHM simulation images are shown in Figure S5. The defect free surface is shown in Figure S5 (a), and as can be seen, the probe particle model correctly shows a series of intersections corresponding to the position of H-Si atoms on the surface. It is important to clarify that the probe particle images show the force of interaction between the tip and surface while the STHM images show the measured tunneling current. As such, the features of these images cannot be directly compared. Since the underlying mechanism of the apparent contrast originates from the same principle, it is still useful to provide a qualitative comparison. It is difficult to compare the DB shown in (b) due to the high amount of current in the experimental images. The Si vacancy in (c) also proves difficult to compare since the structure of the Si vacancy ball and stick model did not distort under the applied molecular dynamics relaxation (see Methods). It can be expected that a more advanced modeling approach which can include charge localization and subsequent lattice distortion will be needed to more accurately match the experimental images of charged species.

The probe particle model does show some success in predicting the structure of the dihydride structures shown in (d), (e), and (f) (3x1, dihydride pair, and single dihydride respectively). Specifically, the model shows the expected distortion of the probe particle over the host dihydride atoms. As discussed previously, the slight variations between the simulated and experimental results are thought to originate from the different signals (force vs. current) measured as well as the unknown apex shape of the experimental tips. The dark and bright missing dimers shown in (g) and (h) also exhibit similar features between the simulated and experimental images. Unfortunately, the STHM images of each were taken with slightly different tips so it is difficult to provide a more detailed comparison. The siloxane dimer in (i) also shows significant similarities between the simulated and experimental images. Specifically, they both show the extension of the dimer length and the distortions (bright features) surrounding the central O atom. The raised SiH_2 and SiH_3 clusters both resemble the form of the Y shape of the SiH_2 and the X shape of the SiH_3, both centered at the position of the raised Si atom. The neutral point defect (which was simulated using a boron atom at the defect point) shows some common features but it is insufficient to confirm whether these originate from our successful prediction of the structure, or an aliasing of features from an entirely different chemical structure.

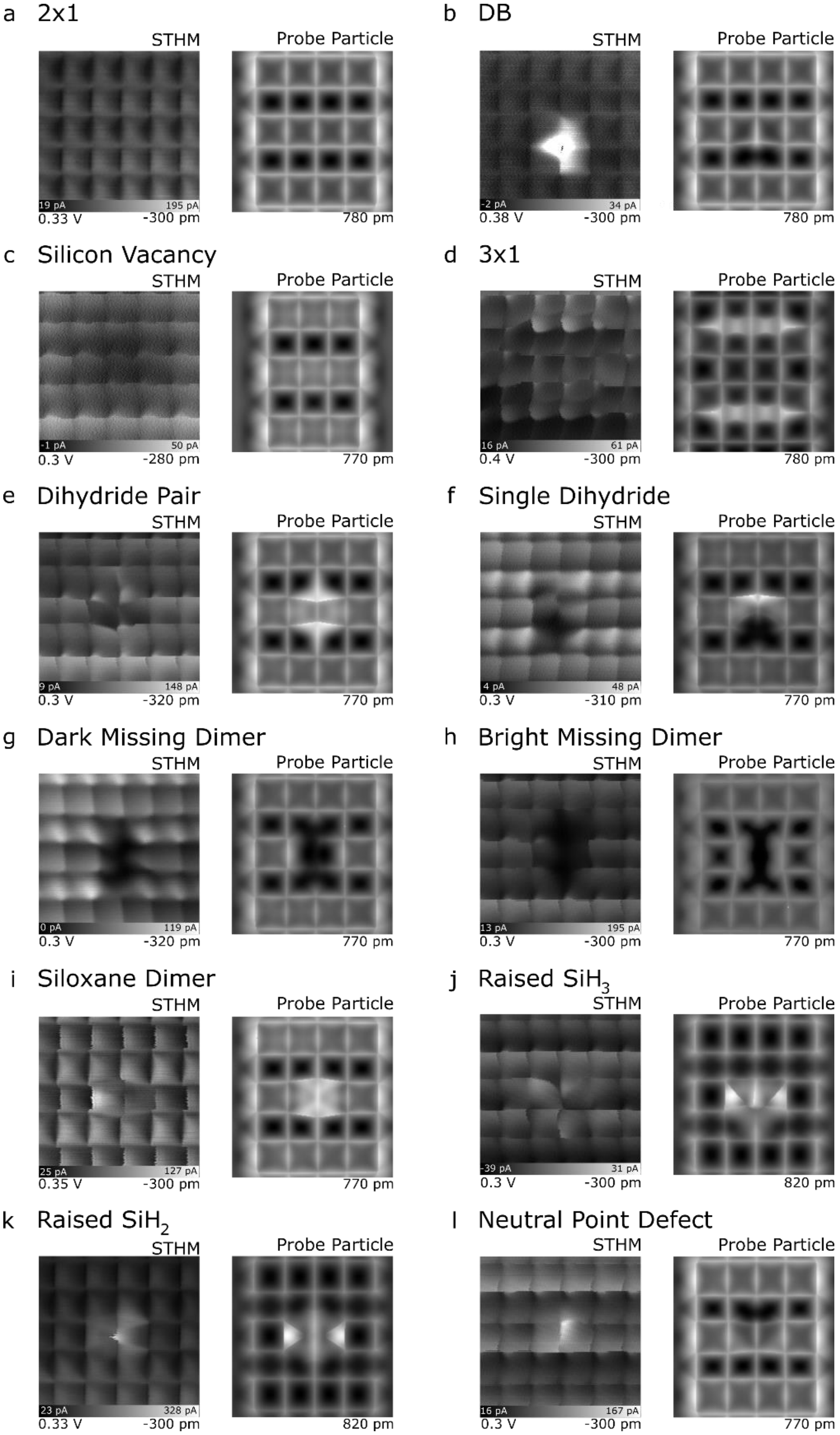

Figure S5. **Simulated STHM images in Comparison with Experiment (excerpt of Figs. 1 and 2)**. The simulated images (right) were selected from a series of simulated heights with a flexible tip apex to best match the contrast in the experimental images (left). The corresponding

distance between the tip and topmost surface Si atom is shown in the lower right of each simulated image. Each image is 2 x 2 nm^2.

The simulated H-apex AFM images are summarized in Figure S6. Here, the experiments and simulated data are related to force. The defect free surface shown in (a) matches reasonably well with the immediate difference being the relative sizes of the H-Si atoms. The H-Si atoms in the experimental images appear to be blurrier in comparison to the simulated images. Since both tips are assumed to have the same size of apex atom (hydrogen), it has been proposed that the difference in surface atom size comes from the flexibility of the surface atoms not modeled in the probe particle model. As shown in previous works[2], at short tip sample separations, the interactions between the tip and sample are strong enough to move the surface atom under the influence of the tip, an effect that is not mimicked by the probe particle model. It can be suspected that this movement of the surface atoms is significant enough to cause the surface atoms to appear larger as they are imaged. In the case of the DB in (b), the simulated image does correctly show the DB as a dark centre but is missing the spherical shape of the DB. As mentioned before, we are hopeful that the inclusion of the DB orbital, charge localization, and relaxation of the DB's silicon atom into an enhanced simulation will account for these differences. The Si vacancy in (c) shows the same deficiencies in predicting the associated lattice distortion with the predicted charge localization. The dihydride species in (d), (e), and (f) (3x1, dihydride pair, and single dihydride respectively) show some success in showing the correct features associated with each dihydride Si atom, however, the experimental results fail to shown the characteristic splitting which is seen in simulated images. As mentioned previously, it is likely that this feature's absence from the experimental images is due to the flexible nature of the surface atoms, an effect which is not captured in the probe particle model. Since the dihydride Si atoms are only held by two Si atoms, it is likely that they are capable of greater flexibility than their 2x1 counterpart. Such a difference could explain the notable deviation between the positions of the inner H atoms in the experimental image when compared to the predicted H atoms of the simulated model.

Nicely reproduced are the features of the dark and bright missing dimers of Figure S6 (g) and (h). Especially in the latter, the neighbouring dimers are simulated to be pulled towards the position of the bright missing dimer in agreement with the experiment. A slight reduction in Δf signal of the H-Si atoms immediately to the right of the missing dimer (the scan direction influences this character) matches a similar reduction in the simulated images. This effect is easier seen in the Si-apex AFM images as will be discussed shortly. The siloxane dimer shows similar features between the experimental and simulated images as well. Both images show an increased distance between the H atoms of the dimer as well as a reduction in signal above the O atom. The effect from the O atom in the experimental image is slightly to the right of the dimer centre due to the scan direction of the image. The reverse scan direction shows the same feature on the other side of the dimer centre. The positions of the raised Si atoms in (j) and (k) relative to the underlying surface align with the simulated images, however, a notable difference is apparent in the shape of the SiH_3 and SiH_2 cluster. Again, this is thought to originate from the model not accurately accounting for flexibility in the surface atoms which would wash out any H features seen in the experimental images. Lastly the neutral point defect in panel l correctly shows a reduction in signal over the atom, but the degree of reduction is much more in the simulated model. This could be due to the model not accurately accounting for any variation in electronegativity or the structural model used being incorrect.

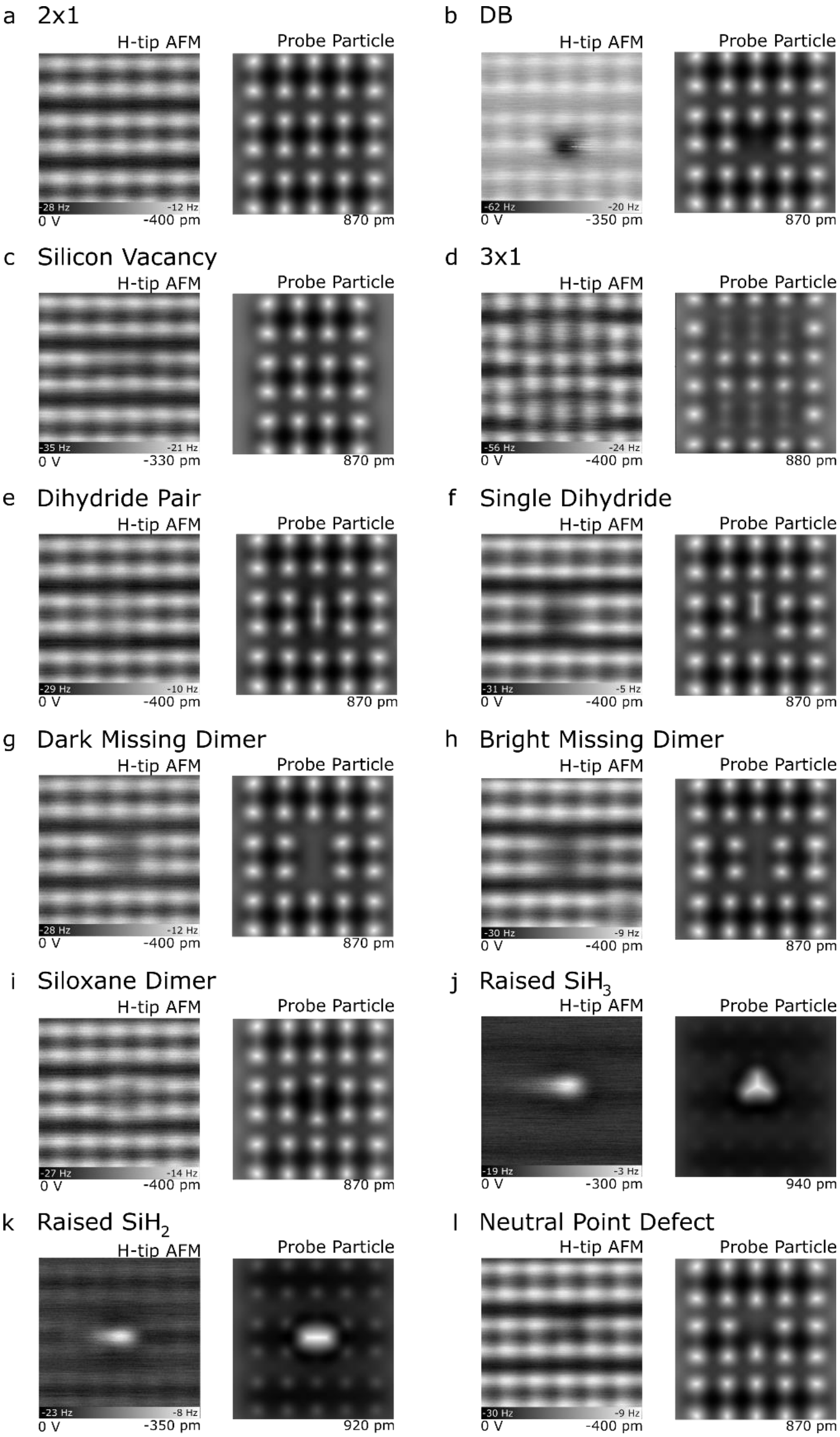

Figure S6. Simulated H-apex AFM Images in Comparison with experiment (excerpt of Figs. 1 and 2). The simulated images (right) were selected from a series of simulated heights with a flexible tip apex to best match the contrast in the experimental images (left). The

corresponding distance between the tip and topmost surface Si atom is shown in the lower right corner of each simulated image. Each image is 2x2 nm^2.

The predicted Si-apex AFM images exhibit the most significant deviation from the experimental results as can be seen for the defect free surface in Figure S7 (a). The characteristic contrast inversion of the H-Si atoms relative to the surface is not present. Since the probe particle model only accounts for a Leonard-Jones type of interaction, it is likely that the unsatisfied orbital of the Si apex atom is contributing an additional covalent like contribution which causes the contrast inversion[5,6]. Similar discrepancies are seen when looking at the DB in (b) and the Si vacancy in (c). An enhanced simulation approach would presumably be able to account for these differences but is beyond the scope of this work. The simulated line profiles of the DB is shown in Figure S11 (t) and further emphasizes the discrepancy between the experimental and simulated interaction.

Despite the difference in contrast between the experimental and simulated results, the probe particle model accurately predicts the atomic positions of the surface atoms as shown in the experimental images. The dihydride species of (d), (e), and (f) (3x1, dihydride pair, and single dihydride respectively) again agree very well with the experimental results. Specifically, the expected splitting of each dihydride species in (d) and (f) is well accounted for. Additionally, the observation of smaller features in the image at the position of the two outer H atoms in the dihydride pair is properly reproduced in (e). Interestingly, the simulated line profile of the dihydride pair in Figure S11 (f) shows the position of the inner two H atoms of the dihydride pair closer together than observed in the experimental line profile. While this difference could be attributed to the slightly asymmetric tip used to image the defect, it is also possible that the molecular dynamics optimization of the surface structure does not accurately capture the observed geometry.

The dark and bright missing dimers of (g) and (h) also show strong resemblance with the experimental images with the simulated image of the bright missing dimer showing a similar reduction in signal in the H-Si atoms immediately surrounding the missing dimer. Such effects are better seen in the line profiles of Figure S11 (j) and (l) which show the reduced interaction at the shoulder of the defect. While the simulation does not fully align with the experimental observation, it is likely that a more robust calculation of the lattice geometry may account for such a difference.

The simulation of the siloxane dimer in (i) agrees well with the experimental images with the two outer H atoms appearing further separated than in a normal dimer as well as the presence of an additional small central feature originating from the O atom (also seen in Figure S11 (h)).

Simulations of both raised Si structures in (j) and (k) show the expected H splitting feature which is absent in the experimental images. While it is possible that structural models for each defect is incorrect, there is also the option for these splitting features to become washed out with the inclusion of surface dynamics in the simulation (line profiles are shown in Figure S11 (n) and (p)).

The neutral point defect of (l) correctly predicts the slight shortening of the dimer length as indicated by the position of the H-Si atom which shares a dimer with the neutral point defect, however, the absence of a stronger signal above the point defect supports the notion that either

the ball and stick model, or the probe particle model contains deficiencies (Line profile is shown in Figure S11 (r)).

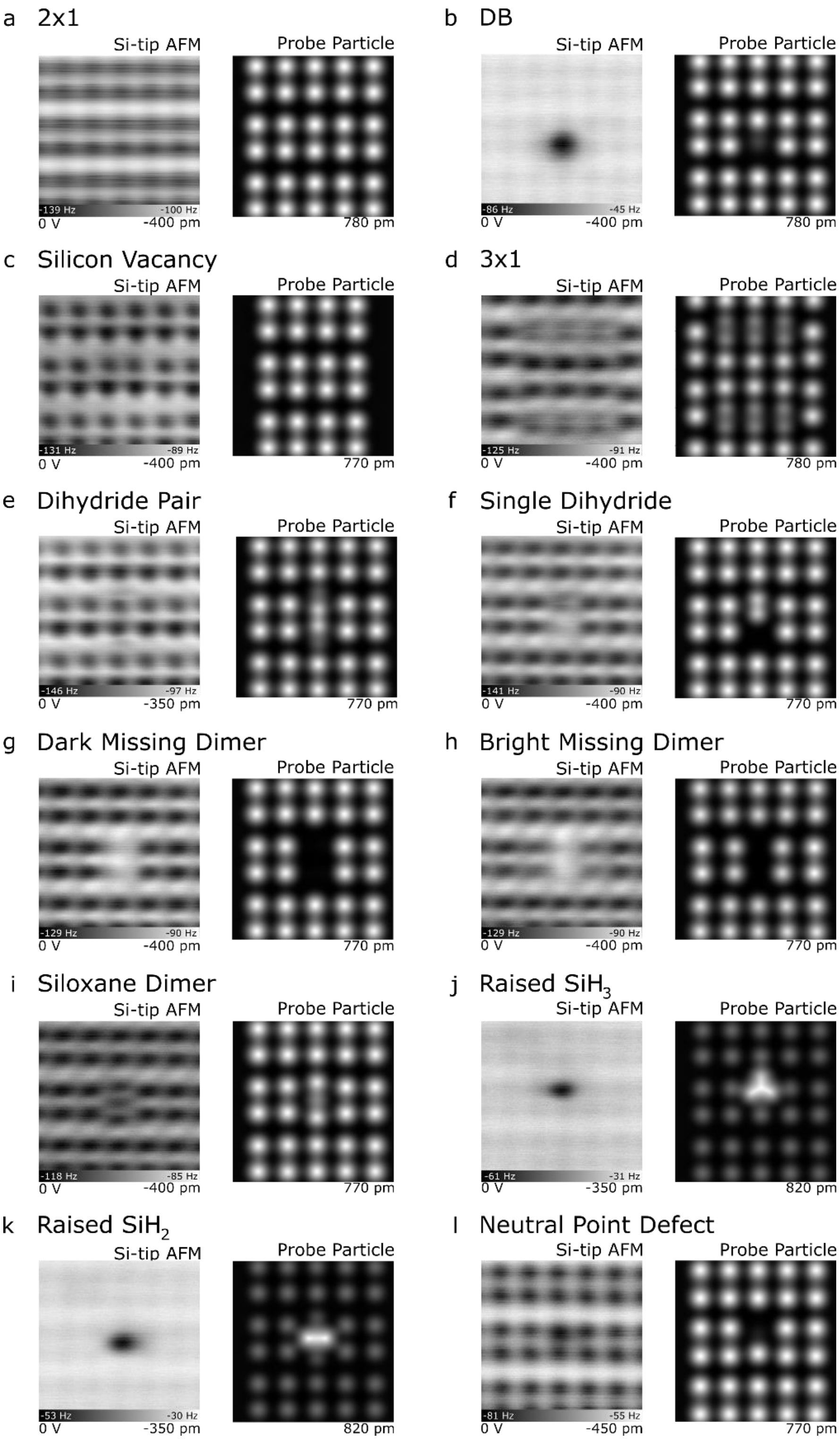

Figure S7. Simulated Si-apex AFM Images in comparison with Experiment (excerpt of Figs. 1 and 2). The simulated images (right) were selected from a series of simulated heights with a rigid tip apex to best match the contrast in the experimental images (left). The corresponding distance between the tip and topmost surface Si atom is shown in the lower right of each simulated image. Each image is 2x2 nm^2.

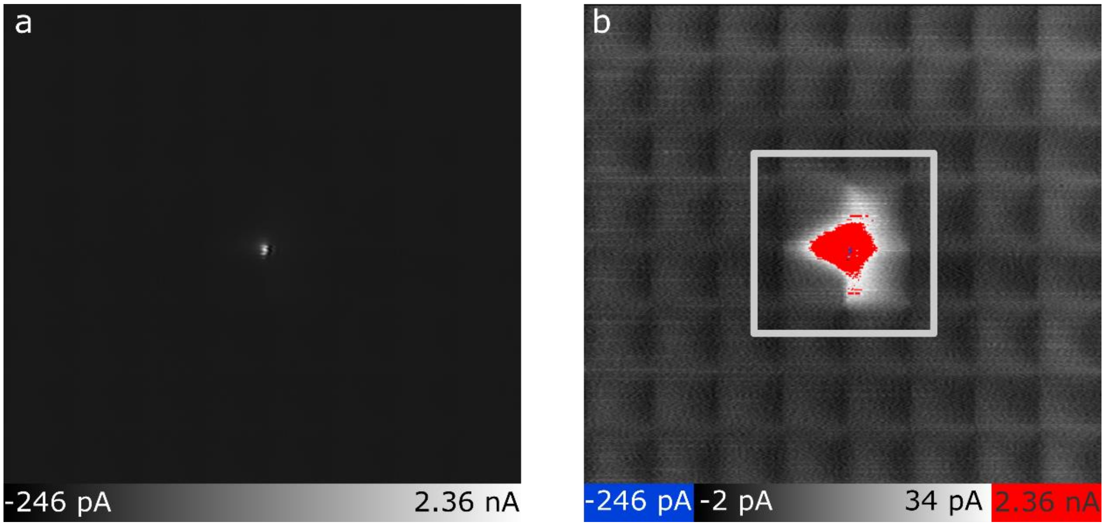

Figure S8. **Full and Saturated Colour Scale Images of the DB in STHM.** (a) Full scale STHM image of the DB from Figure 2a-4, with extremely localized current over the DB (constant height -300 pm, applied bias -0.38 V, image size 3 x 3 nm^2). (b) Color saturated scale of the same image to highlight the H-Si atoms of the surface relative to the DB. The increased and localized nature of the current signal compared to that in Figure 2a-3 is a result of two properties of the H functionalization: the flexibility of the H atom at the apex and its covalent interaction with the bare Si atom of the DB. As the STHM tip scans over the DB, the increase in interaction between the surface DB and the tip leads to significant overlap of the atomic orbitals with the corresponding increase in tunneling current. Few data points close to the centre of the DB show a reversal of measured current, which we attribute to an electronics sampling error.

Raised Silyl Groups

The assignment of the raised SiH_2 and SiH_3 groups comes from the observation of similar imaging characteristics in the empty and filled states images of step edges. Figure S9 (a) shows the filled states image of a step edge with the brighter portion of the image corresponding to a terrace one atomic layer higher than the terrace in the bottom right.

As can be seen, the dimer directions at each terrace are perpendicular to each other with a row of single dihydride groups on the lower terrace due the expected lattice mismatch. The key comparison which lead to the raised SiH_2 and SiH_3 groups being assigned as such is the observation of a dark border at the boundary of the step edge and the rounding of the dimer ends best seen on the two dimers of the upper terrace in Figure S9 (a). The empty states image in Figure S9 (b) shows a different area of the surface but with a similar step edge

configuration. Here, the end of the two dimer rows appear slightly larger and brighter than the remaining atoms of the higher terrace.

The characteristic dark border at the edges of terraces is also seen on smaller structures such as the ones shown in figure S9 (c). Based on their size and position relative to the underlying surface, these are thought to each be two raised Si atoms which have formed their own dimer bond. If we assume that all bonds are saturated with H or neighbouring Si atoms, each structure would have an H-Si-Si-H composition. The observed dark border and rounding of the bright center strongly aligns with the observations made at larger step edges. Similarly, the empty states image in (d) matches those features observed at the terrace edge in (b). The constant height STM image in (e) shows the onset of an STHM contrast due to the reduction in tip sample separation over the defect. Finally, the Si apex AFM image in (f) shows a different version of the same defect where two distinct dark centres appear. By removing one of the two Si atoms, and fully saturating with hydrogen, the resulting structure would be a raised SiH_2 group that sits between two atoms of the same dimer below.

The raised Si group shown in Figure S9 (g) and (h) show the same characteristic dark border in filled states and a similar enhanced brightness in empty states. Based on the defect position with respect to the underlying lattice, this defect appears to be bonded to two Si atoms of neighbouring dimer rows. If we assume that all bonds are fully saturated with Si or H, the structure of the defect would be H_2-Si-Si-H_2. If we remove one of these Si atoms, and fully saturate with hydrogen, the remaining structure would match that of an SiH_3.

Drawing these comparisons between the features seen at a step edge and those seen on smaller structures shown in Figure S9 is what lead to the classification of the raised SiH_2 and SiH_3 defects. The obvious concern to this classification is the absence of any additional hydrogen splitting in a Si apex AFM image. As mentioned in the main text, it is proposed that the absence of such features is due to the enhanced flexibility of the raised Si groups. Since this claim cannot be further supported with more experimental work, additional theoretical work is needed which incorporates such mobility of surface atoms.

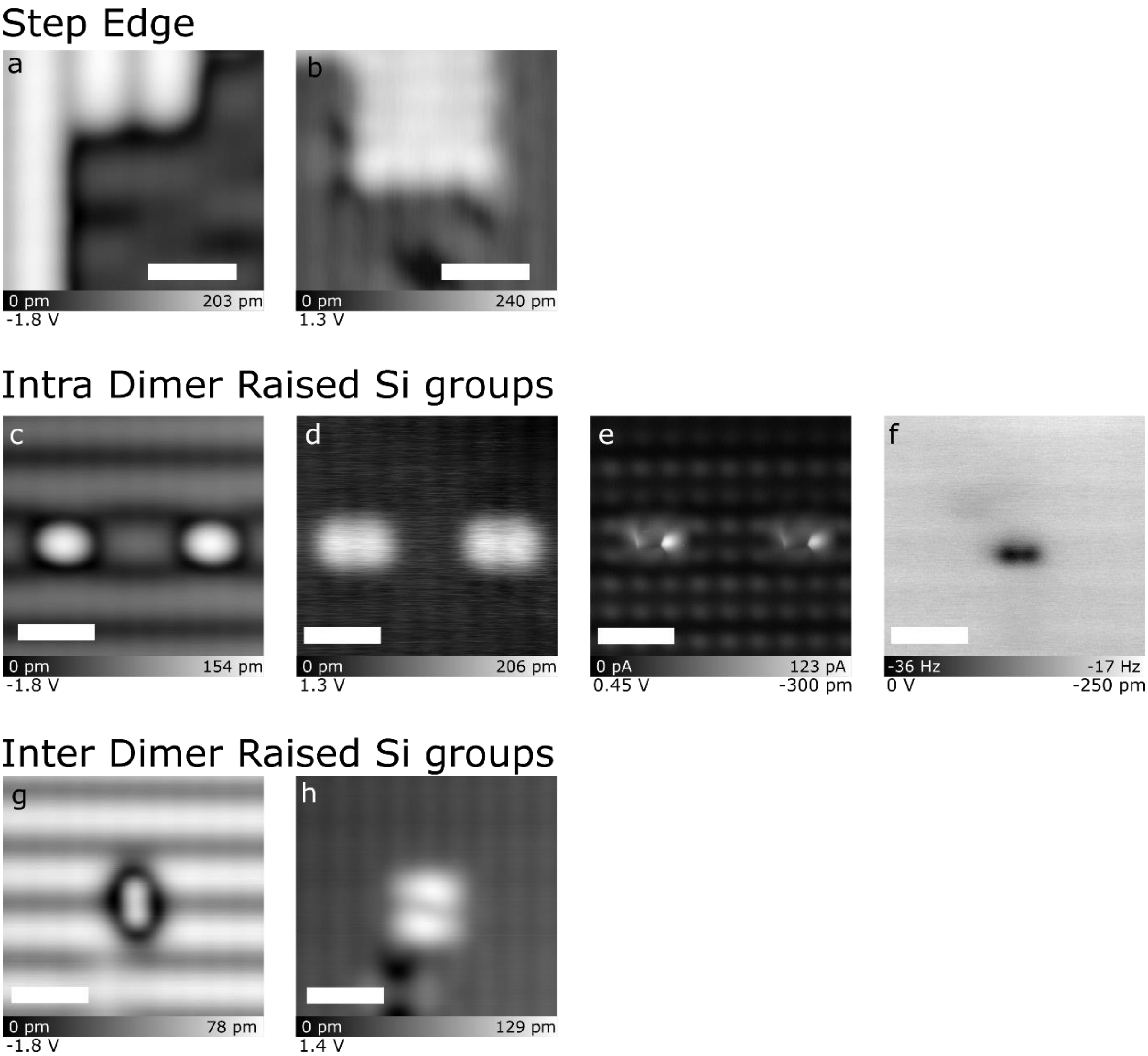

Figure S9. Common characteristics between Step Edges and Raised Si Clusters. (a) Filled states image of a step edge. (b) Empty states image of a step edge taken at a different surface location than (a). (c)-(e) Filled states, empty states, and constant height STM images of a larger raised Si cluster. (f) Si apex AFM image of the same cluster in (c)-(e) at a different spot on the surface. (g),(h) Filled and empty states image of a different raised Si cluster. Imaging parameters are indicated below each image. All scale bars are 1nm.

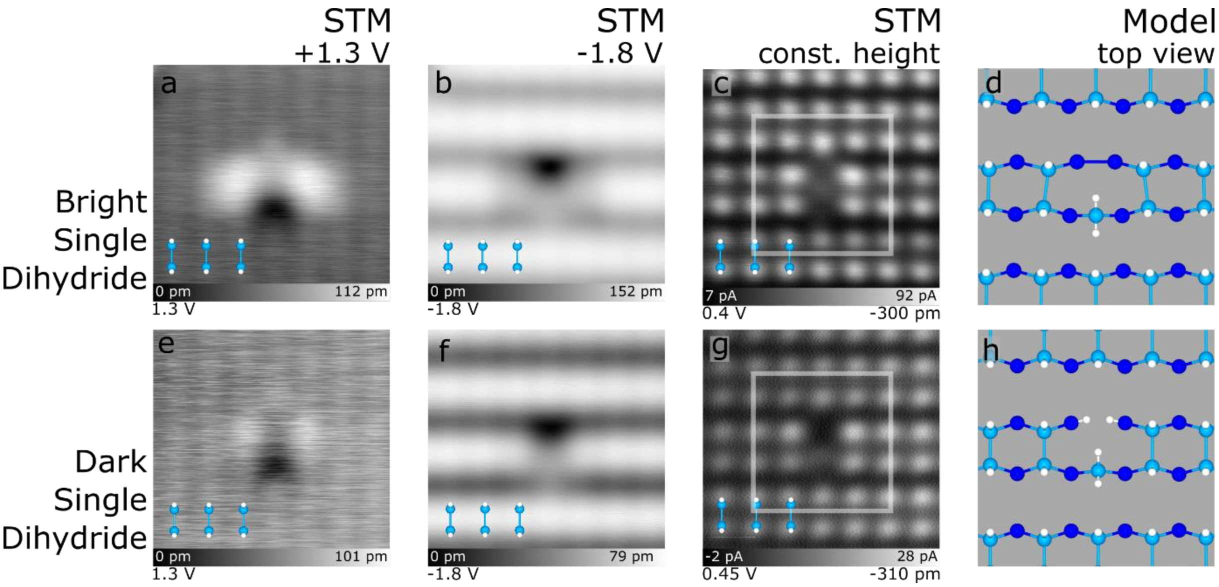

Figure S10 Comparison between bright (top row, a-d) and dark (bottom row, e-h) variation of the single dihydride-missing atom defect. Each image is 3.2 x 3.2 nm^2. All images were taken shortly after one another while carefully monitoring for tip changes. The additional noise in (e) is associated with the telegraph noise of a nearby DB.

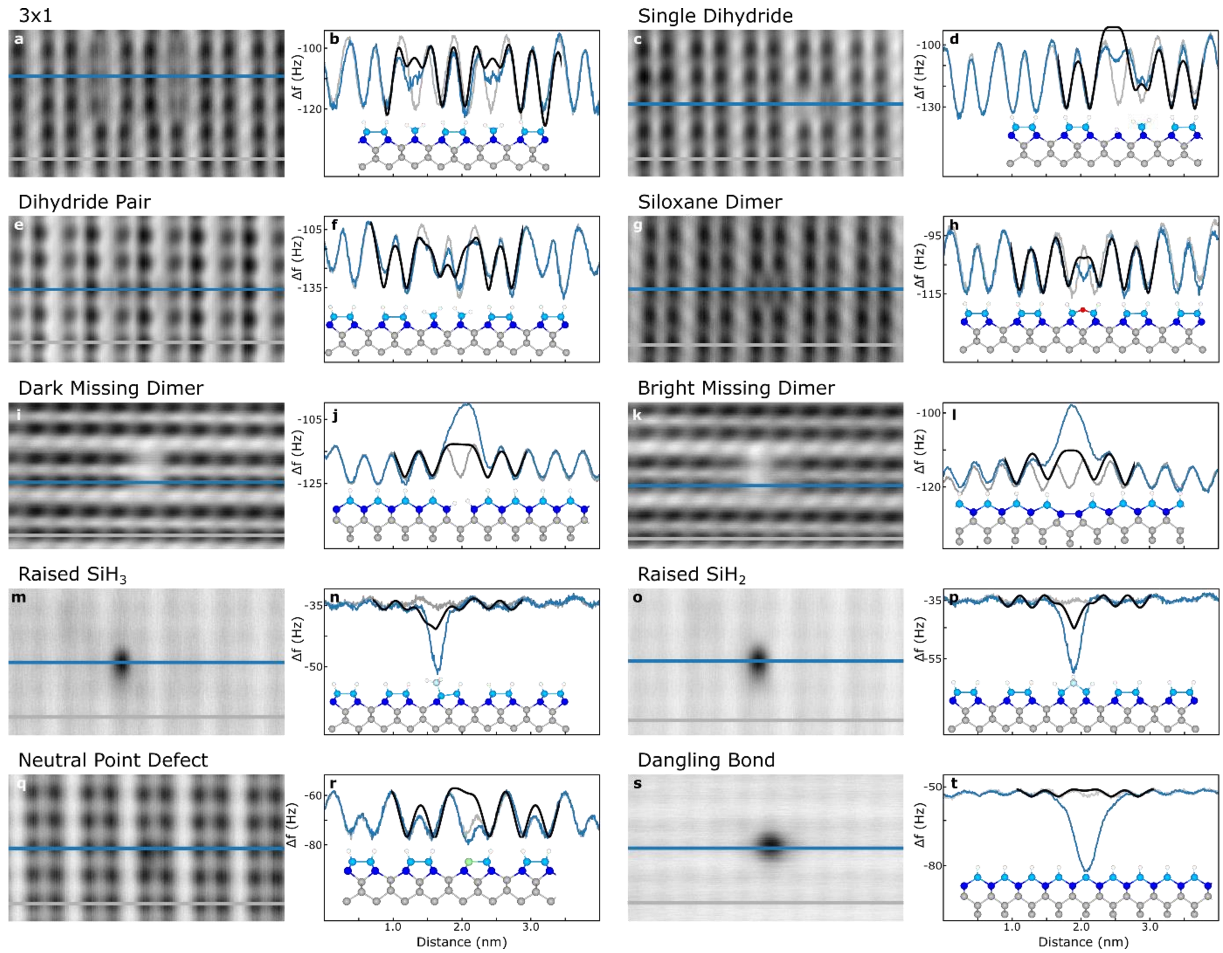

Figure S11. Analysis of Defects using Si-Tip Dark-Contrast AFM and Profile Extractions with the Probe Particle Model. (a)-(s) The same images (left) and line profiles (right) presented in figure 3 with line profiles from the Probe Particle model added in black. Simulated line profiles were taken from the simulated AFM images with a rigid tip. Profiles were inverted and rescaled to best match the experimentally observed atom positions.

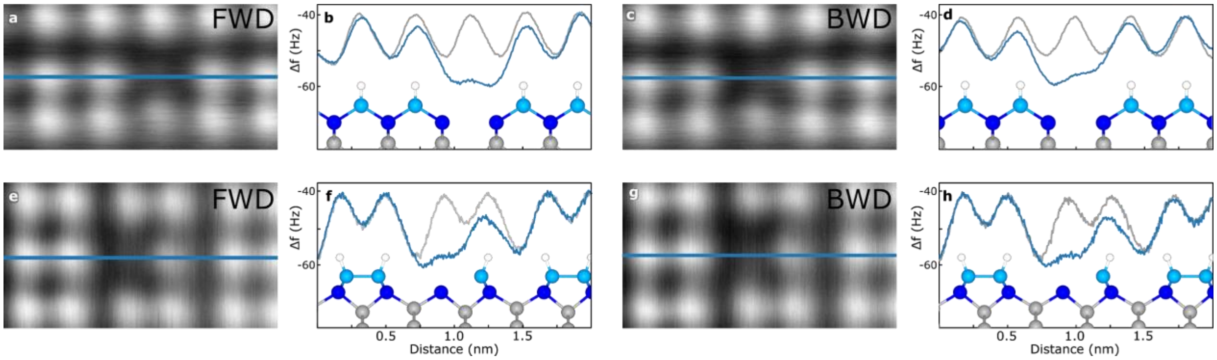

Figure S12. Each of the panels shows a line profile across the Si vacancy "I" (Figure 4a) imaged with H-functionalized bright contrast AFM. Direction of scan for each panel as indicated. The asymmetries in the minima at the vacancy site are likely due to a slight multi-tip made more evident by the small tip sample separation (-400 pm).

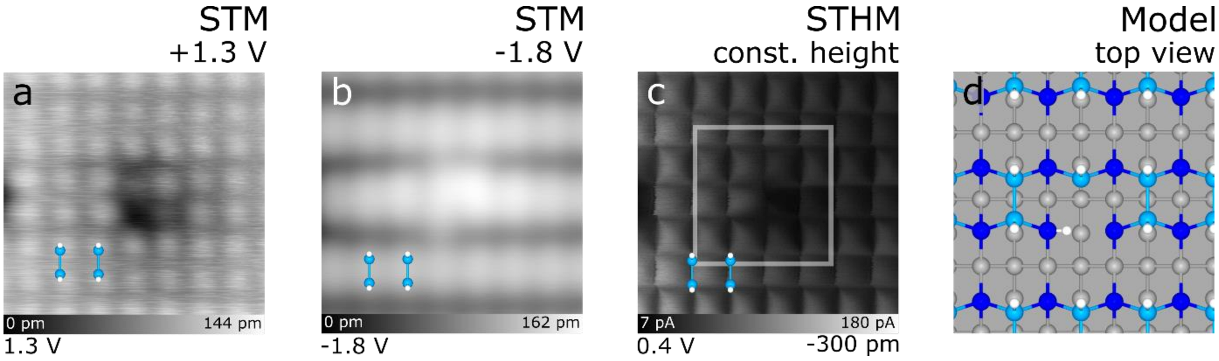

Figure S13. A possible variation of the silicon vacancy I presented in Figure 4. With the addition of hydrogen to the chamber during sample preparation (See Methods), it is suspected that some of the hydrogen radicals can penetrate a few monolayers into the surface, allowing them to passivate dangling bonds from Si atoms immediately surrounding a vacancy[7,8]. Evidence of this is suggested by the vacancy shown here being similar to the one shown in Figure 4a, but with a few key differences. To first order, both display a dark depression on one side of a dimer indicating a missing silicon atom. Differences become apparent however when examining adjacent lattice sites to the missing atom. In the empty states STM image of Figure 4a, the surface hydrogen atoms around the vacancy location have similar character, displaying as darker than the surrounding surface. This is in contrast to Figure S13a where H-Si atoms to the left of the vacancy appear unperturbed, and hydrogen atoms above and to the right are of differing character due to the presence of charge from the remaining DBs. Further differences in the appearance of this configuration are also seen in the filled state STM image of S13b when compared to 4b, with 4b having two maxima and S13b only one. The normal appearance of the surface immediately to the left in S13b supports the idea of a hydrogen penetrating the surface satisfying the left-most sub-surface dangling bond associated with the vacancy (See Figure S13d), reducing the lattice strain and making the surface hydrogen above it better match the defect-free surface. Figure S13c shows an STHM image of the vacancy. This imaging method was used instead of a constant height image due to the tip functionalization at the time of

imaging. Similar features consistent with the interpretation of the vacancy can be observed, mainly, the absence of “corners” in the STHM image above the vacancy as well as a slight negative shift in the current above the lattice intersections corresponding to the upper and right H-Si atoms.

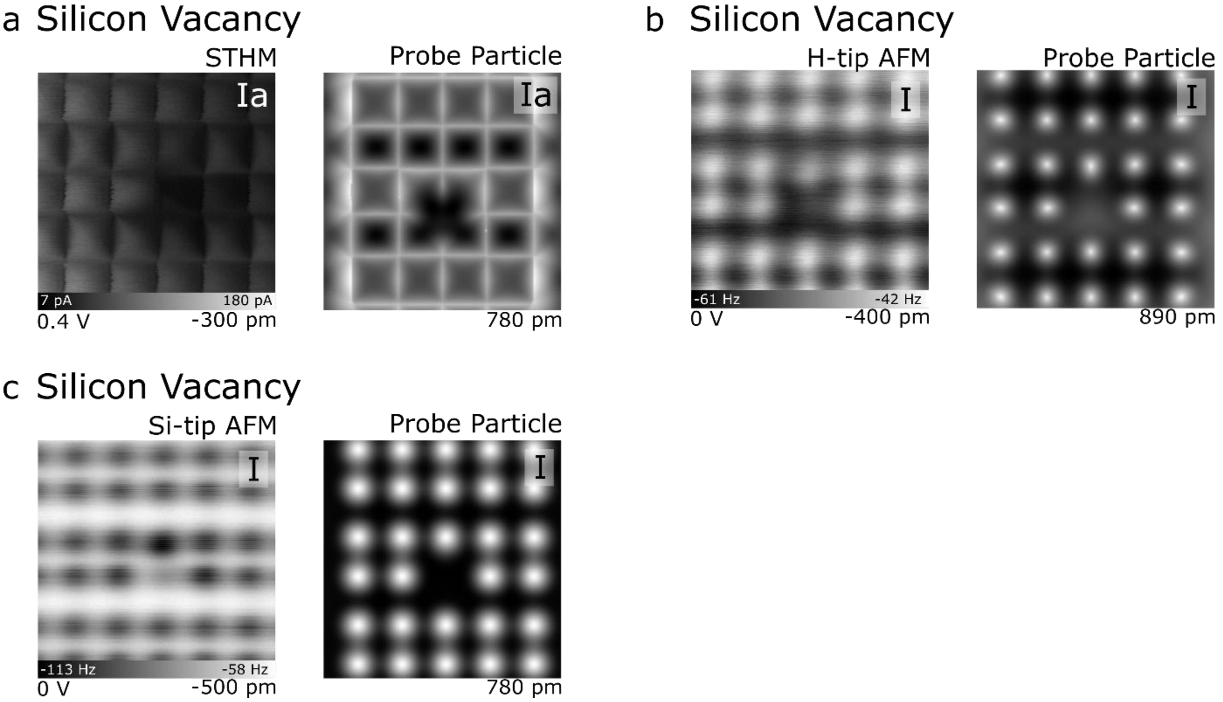

Figure S14. Probe Particle Simulations of the Si Vacancy. (a) STHM image of the Si Vacancy “Ia” (left) and the corresponding simulated image using the Probe Particle Model (right) with a flexible tip apex. (b) H-apex AFM image of the Si Vacancy “I” (left) and the corresponding Probe Particle Model image with a flexible tip apex (right). (c) Si-apex AFM image of the Si Vacancy “I” (left) and the corresponding Probe Particle Model image with a rigid tip apex (right). Heights for the probe particle images are the absolute separation between the topmost surface Si atoms and the metal tip. Each image is 2x2 nm^2.

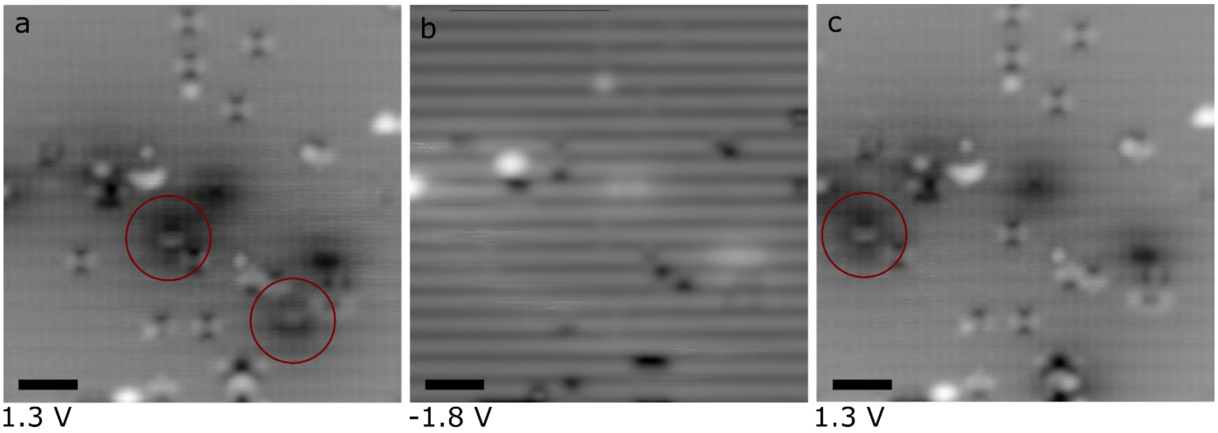

Figure S15. Adsorbed hydrogens. Two hydrogens are seen in (a) (red circle). (b) By scanning at -1.8V, the hydrogens are moved across the surface. This shows as streakiness in the image seen in the bottom left quadrant of the image. (c) Imaging the surface in empty states again, it can be seen one hydrogen shifted to the left side of the image and the other left the scan frame entirely. Scale bar is 2 nm.

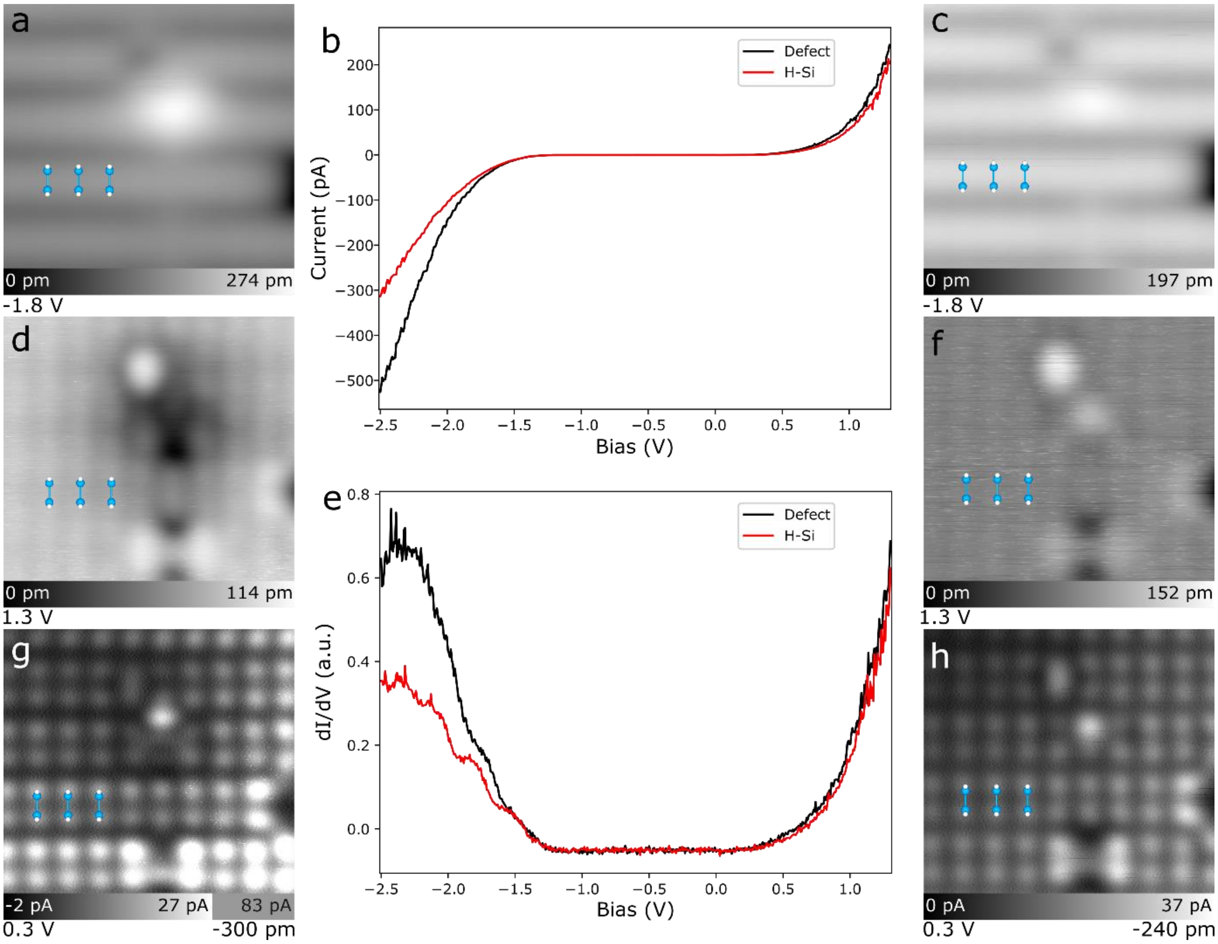

Figure S16. Another example of the H removal from a point defect seen in Figure 5. This time, the scanning bias of -1.8V was not enough to ionize it, so an I(V) spectroscopy was done to probe higher bias values. The starting bias of the spectroscopy was -2.5 V so it is assumed the defect neutralized as the bias was increasing from -1.8V to -2.5V and as such, no neutralization features are seen in the curve. (a,d,g) show the defect in the negative state. (b,e) the corresponding I(V) and dI/dV spectroscopies. (c,f,h) show the neutral variation. Each image is 3.8 x 3.8 nm^2 with imaging parameters as indicated.

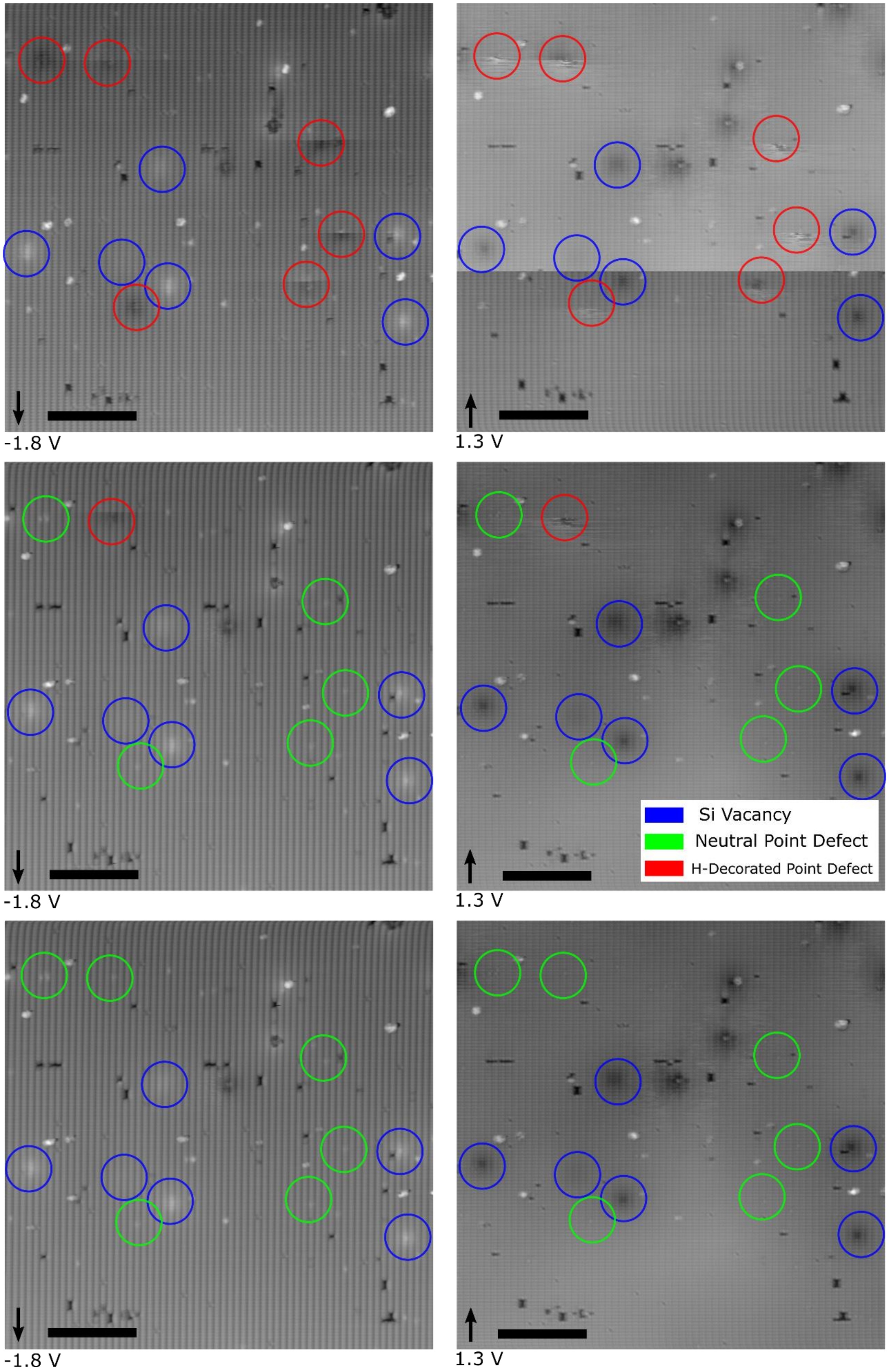
-1.8 V
1.3 V
-1.8 V
1.3 V
Si Vacancy
Neutral Point Defect
H-Decorated Point Defect
-1.8 V
1.3 V

Figure S17. Hydrogen removal from H-decorated point defects. This was taken immediately after an approach before tip conditioning occurred so the tip apex is highly asymmetric. As images are taken at alternating bias values (-1.8V to 1.3 V), one can observe the removal of H from the defect and the associated transition from the negative (red circle) to neutral charge state (green circle). Silicon vacancies are highlighted as well (blue circle) to provide a reference to the charge state of the defects. Scan direction is indicated by the arrows. Scale bar is 10 nm.

<u>References</u>

(1) Hapala, P.; Kichin, G.; Wagner, C.; Tautz, F. S.; Temirov, R.; Jelínek, P. *Phys. Rev. B: Condens. Matter Mater. Phys.* **2014**, *90*, 1–9. doi:10.1103/PhysRevB.90.085421

(2) Labidi, H.; Koleini, M.; Huff, T.; Salomons, M.; Cloutier, M.; Pitters, J.; Wolkow, R. A. *Nat. Commun.* **2017**, *8*. doi:10.1038/ncomms14222

(3) Halgren, T. A. *J. Comput. Chem.* **1996**, *17*, 490–519. doi:10.1002/(SICI)1096-987X(199604)17:5/6<490::AID-JCC1>3.0.CO;2-P

(4) Hapala, P. Probe Particle Model. 2014. https://github.com/ProkopHapala/ProbeParticleModel

(5) Sang, H.; Jarvis, S. P.; Zhou, Z.; Sharp, P.; Moriarty, P.; Wang, J.; Wang, Y.; Kantorovich, L. *Sci. Rep.* **2014**, *4*, 1–12. doi:10.1038/srep06678

(6) Foster, A. S.; Gal, A. Y.; Gale, J. D.; Lee, Y. J.; Nieminen, R. M.; Shluger, A. L. *Phys. Rev. Lett.* **2004**, *92*, 4. doi:10.1103/PhysRevLett.92.036101

(7) Roberson, M. A.; Estreicher, S. K. *Phys. Rev. B* **1991**, 43, 1555-1575. doi:10.1103/physrevb.49.17040

(8) Xu, H. *Phys. Rev. B* **1992**, *46*, 1403–1422. doi:10.1103/PhysRevB.46.1403
